# Taking stock of 10 years of published research on the ASHA programme: examining India’s national community health worker programme from a health systems perspective

**DOI:** 10.1186/s12961-019-0427-0

**Published:** 2019-03-25

**Authors:** Kerry Scott, Asha S. George, Rajani R. Ved

**Affiliations:** 1 Independent consultant, Bangalore, India; 2 School of Public Health, University of the Western Cape, Robert Sobukwe Road, Bellville, Cape Town, 7535 South Africa; 3 National Health Systems Resource Centre, New Delhi, India

**Keywords:** Community health worker, Accredited social health activist, India, health policy and systems research, training and supervision, primary healthcare

## Abstract

**Background:**

As India’s accredited social health activist (ASHA) community health worker (CHW) programme enters its second decade, we take stock of the research undertaken and whether it examines the health systems interfaces required to sustain the programme at scale.

**Methods:**

We systematically searched three databases for articles on ASHAs published between 2005 and 2016. Articles that met the inclusion criteria underwent analysis using an inductive CHW–health systems interface framework.

**Results:**

A total of 122 academic articles were identified (56 quantitative, 29 mixed methods, 28 qualitative, and 9 commentary or synthesis); 44 articles reported on special interventions and 78 on the routine ASHA program. Findings on special interventions were overwhelmingly positive, with few negative or mixed results. In contrast, 55% of articles on the routine ASHA programme showed mixed findings and 23% negative, with few indicating overall positive findings, reflecting broader system constraints. Over half the articles had a health system perspective, including almost all those on general ASHA work, but only a third of those with a health condition focus. The most extensively researched health systems topics were ASHA performance, training and capacity-building, with very little research done on programme financing and reporting, ASHA grievance redressal or peer communication. Research tended to be descriptive, with fewer influence, explanatory or exploratory articles, and no predictive or emancipatory studies. Indian institutions and authors led and partnered on most of the research, wrote all the critical commentaries, and published more studies with negative results.

**Conclusion:**

Published work on ASHAs highlights a range of small-scale innovations, but also showcases the challenges faced by a programme at massive scale, situated in the broader health system. As the programme continues to evolve, critical comparative research that constructively feeds back into programme reforms is needed, particularly related to governance, intersectoral linkages, ASHA solidarity, and community capacity to provide support and oversight.

**Electronic supplementary material:**

The online version of this article (10.1186/s12961-019-0427-0) contains supplementary material, which is available to authorized users.

## Introduction

National community health worker (CHW) programmes are receiving renewed attention as mechanisms to help overcome health worker shortages, retain health workers in underserved areas, and provide culturally appropriate primary healthcare [1–3]. No longer seen as stop-gap or a temporary solution, they are now considered as part of long-term investments for responsive and community-oriented health systems [4, 5].

Despite such endorsement, several gaps in knowledge remain in ensuring that CHW programmes deliver at scale across diverse national contexts. There is ongoing discussion about how best to facilitate and sustain community and health systems support for CHW programmes [5], how CHWs should be remunerated [6–8]. and the role of CHW programmes in supporting or undermining health worker rights and gender equity [9–11]. Looking to the future, questions arise on how to support CHW programme evolution to meet changing health needs (e.g., increasing prevalence of non-communicable diseases), changing contexts (e.g., rapid urbanisation), and changing roles (e.g., engaging in service accountability) [12]. Reviews of the literature on CHW programmes have provided valuable insight into health system considerations of CHW programmes [3, 13, 14], including on supervision strategies [15, 16], the influence of context and programme features on CHW productivity [17–19], the extent to which CHW programmes provide equitable healthcare [20], cost effectiveness [21], and considerations for operating national, scaled up programmes [22, 23]. Country-specific literature reviews have been performed for Brazilian [24–26] and Ghanaian [27] CHW programmes; these reviews identified policy and research gaps and explored how CHW roles and identities bridge the gap between community and health services. The only existing review on CHW programmes in India focuses on the rights of CHWs themselves [9], identifying shortcomings in terms of remuneration and labour rights.

In 2005, India launched the accredited social health activist (ASHA) programme as a key component of their National Rural Health Mission to strengthen rural government service delivery, as well as community engagement and ownership in health programmes [28]. The ASHA programme involved the selection of one woman per village (approximately 1 per 1000 population) who would receive an initial 23 days of training on basic health topics and link community members to health services, provide basic first aid and supplies, and mobilise the community around water, sanitation, nutrition and health issues. In 2015, the programme matured into the National Health Mission and was extended to marginalised urban areas. With almost one million ASHAs now selected and trained, it has grown to become one of the largest CHW programmes in the world. As it enters its second decade, we take stock of the current knowledge base understand the nature of the research undertaken and whether it examines the health systems interfaces required to sustain and evolve such a large-scale programme.

## Methods

Systematic mapping – rather than systematic review – was appropriate for our interest in identifying and describing all articles published on the topic. Systematic mapping enables the inclusion of the entire range of academic research on the topic of interest, rather than limiting inclusion to research that addresses a single, clearly defined systematic review question [29]. Since systematic mapping includes articles using any methodology, and since it seeks to define the landscape of work on the subject rather than present evidence on a specific narrow question, assessment of the quality of included research is not appropriate.

### Search strategy

We searched the electronic databases PubMed, Embase and Scopus, which index the largest number of publications and most prominent journals in public health, biomedicine and the social sciences [30–32], for articles published between 1 January 2005 and 9 August 2016. The year 2005 was selected as the beginning date because that was when the ASHA programme was announced and launched. Searches were developed in consultation with an academic librarian at Johns Hopkins University. Searches incorporated keywords and free text for two concepts, namely CHWs (e.g. global terms such as ‘community health worker’ and ‘lay health worker’ as well as ‘accredited social health activist’, ‘ASHA’ and the state-specific names of the ASHA programme, such as *Mitanin* and *Sahyogini*) and India (e.g. India* and the names of all Indian states), with the two strings joined by the Boolean operator ‘AND’. See Additional file 1 for the full search strategy.

### Eligibility criteria, screening and article selection

Articles were included if they (1) presented substantial information on India’s ASHA CHWs and (2) were academic empirical research or commentaries. We included articles that discussed multiple CHW programmes as long as they had meaningful content on the ASHA programme. We included full text manuscripts as well as published abstracts and short articles. We excluded research protocols as well as articles that did not directly mention ASHAs or mentioned them only in passing without information on their role. We also excluded articles about Indian CHW programmes that were not the government’s ASHA programme (e.g. smaller NGO programmes). One author reviewed all titles and abstracts. Potential bias was mitigated in two ways. First, the reviewing author took an inclusive approach in terms of article format and content, accepting article formats including research abstracts and commentaries, and only excluding articles that clearly had no meaningful content on the ASHA programme. Second, all borderline or unclear cases were discussed with the other authors to reach consensus on inclusion. Full texts of retained articles underwent a final screening for eligibility. We were willing to include articles in Hindi; however, no non-English articles were identified. Grey literature on the ASHA programme was beyond the scope of this review, as we were seeking to understand and synthesise the current academic research published on the programme.

### Data extraction and synthesis

Detailed data were extracted by one researcher into a pilot-tested framework in Microsoft Excel, which included the following data extraction components and article assessments.

#### Location

We assessed the location of the research conducted on the ASHA programme to describe its geographical spread (presented in Fig. 3). To do so, we counted the number of studies in each Indian state. Studies conducted in multiple states were counted once for each state. We did not count articles that took a national perspective or that did not specify the state where their research was conducted.

#### Health condition focus

Articles that focused on a particular health condition were classified into the following categories: neonatal and child health, sexual and reproductive health, communicable diseases, and non-communicable diseases (Table 1). Any article that did not have a specific health condition focus was classified as ‘general ASHA work’.

#### Routine versus special intervention

We classified all articles according to whether they focused on the routine work of ASHAs within the national programme (e.g. assessments of typical ASHA practice) or were small-scale special interventions that engaged with ASHAs above and beyond their routine government work (e.g. pilot mHealth interventions or special surveys that used ASHAs as enumerators) (also presented in Table 1).

#### Evaluation outcomes

Among studies that presented evaluative findings, we assessed whether these findings were broadly positive (such as ASHAs having high knowledge or effectively performing a new skill), negative (such as ASHAs having poor knowledge or low motivation) or mixed (such as ASHAs facing significant challenges but also gaining self-esteem, effectively delivering some health services or benefitting from key support structures).

#### Health systems perspective

We considered an article to have taken a health systems perspective if it examined health systems elements, such as supervision, training, supply chain management, financing, motivation, etc., or if the article discussed linkages or repercussions between health systems dimensions such as how communities supported ASHAs or whether facility providers were responsive to ASHAs.

#### CHW–health systems interface

**Fig. 1:**
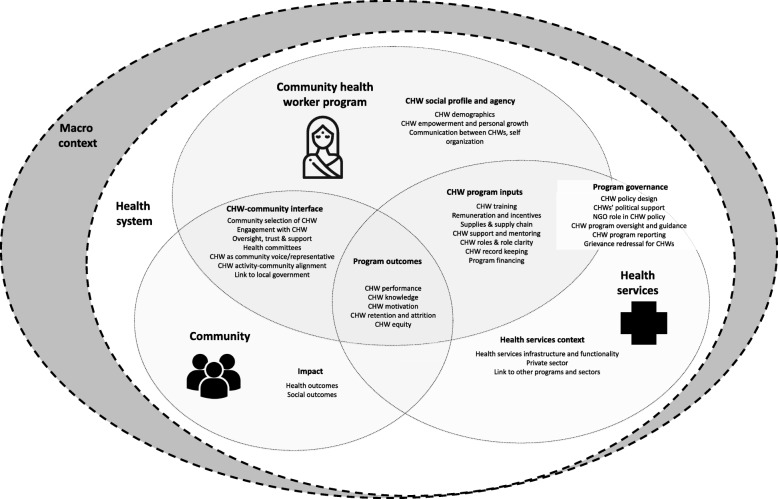
Community health worker – health system interface framework

We developed a CHW–health system interface framework and assessed the number of publications providing information on the topics in the framework, specifically on CHW social profile and agency, CHW programme inputs, CHW–community interface, health services context, programme governance, programme outcomes, and programme impact (Fig. 1). The framework was developed through discussion among team members, review of existing CHW frameworks [5, 18, 19, 23, 33, 34], and during a workshop among Indian and African researchers. While reading each article, we extracted any meaningful content falling under each of the topics in the framework and then counted the number of articles providing information for each topic. For an article to be counted as providing meaningful information it had to present some new data or novel comment, critique or interpretation related to the topic. Thus, for example, under the topic ‘CHW programme inputs’ sub-topic ‘remuneration and incentives’ an article presenting a background description of the remuneration system for ASHAs would not be counted, whereas an article pilot testing an add-on incentive or presenting findings on how ASHAs feel about their remuneration would be counted. Table 2 presents details on how each topic applies to the ASHA programme and the number of articles with substantive content on each topic.

#### Research typology and methodology

We classified each study according to whether they were reflective commentaries or primary research. All primary studies were further classified according to a health policy and systems research typology [35], wherein ‘descriptive research’ describes a context, population or phenomena, enabling comparability with other contexts and experiences; ‘exploratory research’ builds initial understanding and early hypothesis; ‘explanatory research’ involves in-depth research often with triangulation and using and testing theory to explain causal mechanisms; ‘research to establish influence’ (adequacy, plausibility or probability) assesses the impact of one variable on another; ‘emancipatory research’ is used to jointly understand a problem, act on it, and learn from working collaboratively to address power; and ‘predictive research’ is used to anticipate the consequences of decisions.

#### Authorship

To analyse authorship, we assessed the institutional affiliation of the first author as domestic (based in India), foreign (any country other than India) or global (international bodies, including WHO, UNICEF and the World Bank). To understand collaboration and partnership between Indian and foreign institutions, we coded the affiliations of all authors as solely domestic (Indian), solely foreign, mixed domestic and foreign, or global.

## Results

**Fig. 2:**
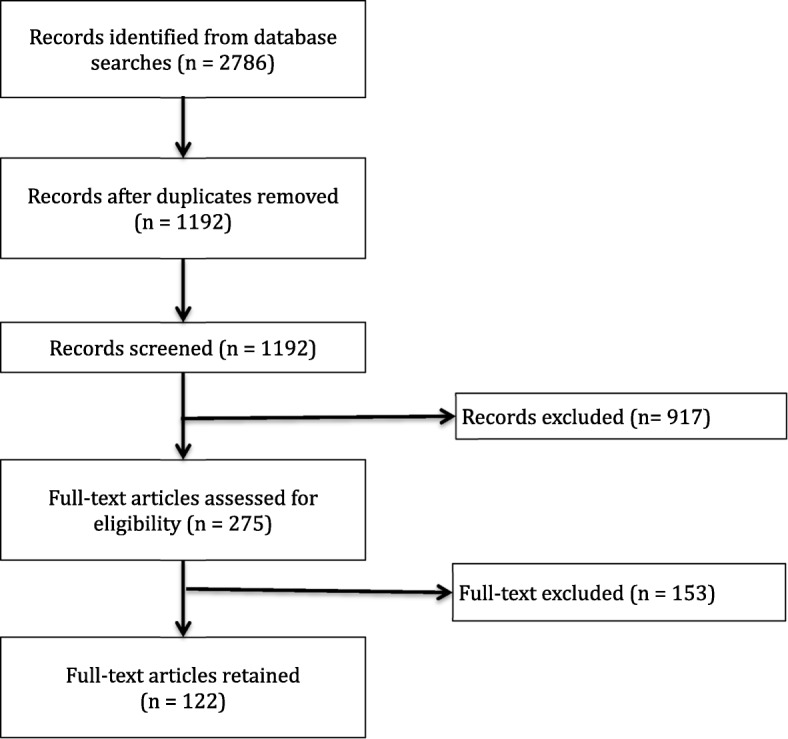
Diagram of article screening process

From 2786 unique references identified in our search, 122 articles met our inclusion criteria (Fig. 2), of which 56 were quantitative, 29 mixed methods, 28 qualitative, and 9 commentary or synthesis. Additional file 2 presents the complete references of included articles. Additional file 3 presents a sortable excel database of included articles. Additional files 4 and 5 provide an overview of article findings.

### Location: where in India is research on the ASHA programme being performed?

**Fig. 3:**
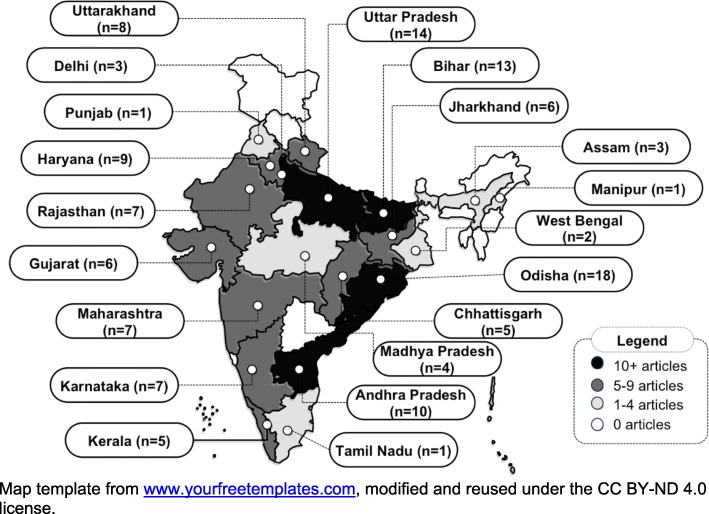
Research on the ASHA programme, by state

Seventeen articles were about the ASHA programme as a national programme and thus did not have a specific research location. Of those articles that specified geographic location, while 13 involved comparative research conducted in multiple states [36–48], the great majority was conducted within a single state. Overall, research on the ASHA programme is concentrated in large northern states (Fig. 3), with most articles reporting research from Odisha (formerly Orissa) [42–44, 47, 49–62], then Uttar Pradesh [36, 38–40, 46, 63–71] and Bihar [36–39, 44, 46, 48, 72–77]. Significant numbers of published articles came from Andhra Pradesh [41, 44, 78–85], Haryana [86–94], Uttarakhand [40, 95–101], Maharashtra [37, 45, 102–106], Rajasthan [37, 40, 44, 45, 107–109], Karnataka [110–116], Jharkhand [42–44, 47, 117, 118], and Gujarat [38, 39, 41, 119–121]. In contrast, fewer articles were published on the ASHA programme in Kerala [44, 122–125], Chhattisgarh [126–130], Madhya Pradesh [131–134], Delhi [135–137], Assam [44, 138, 139], West Bengal [44, 48], Manipur [140], Punjab [141], and Tamil Nadu [37].

There is no research from the union territories,^1^ which are administrative areas with no legislative assemblies and have very few ASHAs. There was also no research from several small north-eastern states (Sikkim, Arunachal Pradesh, Meghalaya, Mizoram, Nagaland and Tripura), Jammu and Kashmir or from Telangana, which was created in 2014 and thus has only existed as a separate entity for a few years.

### Health condition focus of articles

Out of 122 published articles, 76 (62%) focused on a particular health condition (Table 1). The most common health foci were neonatal and child health (*n* = 26) and sexual and reproductive health, including maternal health (*n* = 26), with fewer articles focusing on communicable (*n* = 13) and non-communicable diseases (*n* = 9). The more affluent southern Indian states of Kerala, Andhra Pradesh and Karnataka were frequently the research sites for innovative efforts to address non-communicable diseases [85, 110, 122, 123, 125]. In addition, all five of the publications on HIV are from one project in Andhra Pradesh on engaging with ASHAs to support women living with HIV [79–83].

Of the 46 articles (38%) without a specific health topic focus, many relate to cross-cutting functions that support the ASHA’s core role as maternal and child health promoters. These articles include research on the association between educational level and ASHA capacity (such as filling in the village health register tracking children and pregnant women) [65], the ASHA’s role in multiple reproductive, maternal and child health functions [50, 51, 56], in village health and nutrition days [59, 99], and in village health, sanitation and nutrition committees [43] (Table 1).

**Table 1:** Health condition focus of publications on ASHAs (*n* = 122)

Health condition focus	Routine	Special	Total	Health policy and systems research
Neonatal and child health			28	11
• Integrated management of childhood illness [86, 88, 90, 156], pneumonia care [36], diarrhoea care [38, 39], other child health [105]	5	4	9	4
• Home-based neonatal care [63, 93, 119, 138], other newborn health [94, 144, 150]	3	5	8	4
• Immunisation [40, 46, 145, 148]	4	0	4	2
• Nutrition (breastfeeding, infant and young child feeding) [97, 106, 115, 129]	1	3	4	0
• Other: childhood developmental delay and disability [124], infant and maternal death tracking [113], pregnancy, delivery and infant death tracking [120]	0	3	3	1
Sexual and reproductive health, including maternal health			27	12
• Maternal healthcare, including pregnancy detection, antenatal care, pre-eclampsia, timely referral [37, 64, 87, 101, 114, 136, 141], institutional delivery [52, 55, 92, 132, 133, 137], maternal death investigation [134]	12	3	15	11
• Abortion and contraceptives [67, 107, 117, 118, 146]	1	4	5	1
• HIV [79–83]	0	5	5	0
• Others: uterine prolapse [95], cervical cancer screening [159]	0	2	2	0
Communicable diseases			13	3
• Malaria [49, 54, 60, 61, 139]	3	2	5	3
• Leprosy [48, 77, 78]	1	2	3	0
• Visceral Leishmaniasis (kala-azar) [72, 73, 76]	1	2	3	0
• Others: tuberculosis [104], filariasis [58]	2	0	2	0
Non-communicable diseases			8	2
• Mental health [91, 110]	2	1	3	0
• •Cardiovascular diseases [85, 125]	1	1	2	1
• Multiple non-communicable diseases [122, 123]	0	2	2	1
• Tobacco control [41]	1	0	1	0
Sub-total of all publications with a health focus	37	39	76	28
General ASHA work	41	5	46	45
**Total**	*78*	*44*	*122*	*73*

### Routine versus special intervention focus

Of the 122 papers, 78 (64%) focused on the routine ASHA programme. Details of these publications are presented in Additional file 4. The remaining 44 (36%) were about special interventions that engaged ASHAs beyond their official roles and responsibilities such as pilot interventions. Details of these programmes are presented in Additional file 5.

### Evaluation outcomes

Findings in the publications on the routine ASHA programme were mostly mixed (55%, *n* = 43/78) and negative (23%, *n* = 18/78), with few indicating overall positive findings (13%, *n* = 10/78). In contrast, findings in the publications on special interventions were overwhelmingly positive (77%, *n* = 34/44), with few negative (5%, *n* = 2/44) or mixed (9%, *n* = 4/44) results. Article-wise assessments of evaluation outcomes can be found in Additional files 4 and 5.

### Health systems perspective

Over half the articles (60%, *n* = 73/122) took a health systems perspective when researching and discussing the ASHA programme (right-hand column of Table 1). While only one-third of the 76 studies with a health condition focus had a health systems perspective (37%, *n* = 28/76), almost all the 46 studies on general ASHA work did so (98%, *n* = 45/46). The only article on general ASHA work that did not take a health system perspective was an assessment of ASHA knowledge across a range of topics related to their expected areas of work [131].

### CHW–health systems interface

When considering article content in relation to the CHW–health system interface framework (Fig. 1), across the 122 articles, ASHA performance (65%, *n* = 79/122) and ASHA training and capacity-building (51%, *n* = 62/122) were discussed across the largest number of publications (Table 2). At the other end of the spectrum, five or fewer articles discussed programme financing, programme reporting, grievance redressal for ASHAs, and communication between ASHAs (Table 2).

**Table 2:** CHW–health system interface topics discussed in publications on ASHAs

Main topic	Sub-topics	Application to ASHA programme	Number of publications with content on sub-topic
Programme inputs	Training and capacity-building	Descriptions of training and capacity-building provided to ASHAs, challenges associated with ASHA training, how ASHAs feel about training, percentages of ASHAs receiving training	62
	Supplies and supply chain	The type and amount of supplies ASHAs receive (including their routine drug kids and extra supplies from small scale interventions, such as mHealth programmes), how ASHAs feel about their supplies, issues of resupply and stock outs	42
	Support and monitoring	ASHA relationships with frontline workers (such as auxiliary nurse midwives, medical officers, ASHA facilitators), including how they work together and the extent to which they receive monitoring and supportive supervision (routinely and in small-scale interventions)	41
	Incentives and remuneration	Financial incentives provided and changes to them, timeliness and access to remuneration, ASHA satisfaction with remuneration	38
	Roles and role clarity	The roles ASHAs have been given, including the evolution of routine roles and roles given in special interventions, and the extent to which ASHAs understand their roles	35
	CHW record keeping	What records ASHAs maintain (such as monthly reports, village registers), how they feel about these records, challenges related to ASHA record keeping such as completeness and correctness	16
Programme outcomes	CHW performance	Functionality, effectiveness, skills, practice – what services ASHAs provide, what tasks they perform, and/or how well they provide these services or perform these tasks, including coverage of services (but not in terms of equity or which households they cover, which is counted in ‘CHW equity’)	79
	CHW knowledge	How much ASHAs know about various health topics	30
	CHW motivation	ASHA job satisfaction, how motivated ASHAs are to do their work, what their sources of motivation are (but not their motivation to continue working as an ASHA, which is counted below under ‘retention and attrition’)	30
	CHW equity	Which households ASHAs reach in terms of marginalisation, social distribution of services	17
	CHW retention and attrition	Intention to remain an ASHA, drop-out rates	9
CHW–community interface	Community engagement with CHWs	Community knowledge of their ASHA, trust in their ASHA and satisfaction with their ASHA, factors that influence this relationship	34
	Resonance of CHW activities	Extent to which ASHA activities align and resonate with community needs	22
	Community selection of CHW	Extent and manner through which the community was involved in deciding who should be their ASHA	16
	Link to local government	Involvement of the local government structure (*panchayat*) in any aspect of the ASHA programme	15
	Health committees	ASHA role in convening village health and sanitation committee meetings, activities undertaken by the committee in relation to the ASHA	13
	Community oversight of and support for CHW	Community oversight of the ASHA, ASHA accountability to the community, community provision of support for the ASHA	10
	CHW as community voice or representative	ASHA as a representative of the community’s perspectives, voices and needs to health system functionaries	8
Programme governance	CHW policy design and development	Policy recommendations to strengthen the ASHA programme, discussions about how policy was set or should be set, critiques of ASHA-related policy	27
	CHW programme oversight and guidance	Formal oversight and guidance systems to shape the ASHA programme, particularly the role of national and state health resource centres	8
	CHW political support	Political buy-in to the ASHA programme at the national and state levels; ASHA political engagement and advocacy, such as through unionisation	7
	Role of NGO actors in CHW policy	Role of NGO actors in shaping the ASHA programme, such as NGOs, academics and private sector interest groups	6
	Grievance redressal for CHWs	The development and functioning of formal government mechanisms through which ASHAs could register grievances	5
	Programme financing	How much money is budgeted to fund the ASHA programme and how these decisions are made, release of funding, comments on financing issues	5
	CHW programme reporting	Systematic programme management records that the government system creates about the ASHA programme (such as national or state level reports, annual nodal officer meeting minutes)	2
CHW social profile and agency	CHW demographics	ASHA demographic information such as ASHA age, caste, marriage, and literacy statistics, or comments on these issues (e.g. caste dominance among ASHAs)	41
	Empowerment and personal growth	Comments or research on ASHA well-being, personal growth, rights-related challenges and opportunities including for leadership, career progression or educational advancement	14
	Communication between CHWs	Opportunities and nature of ASHA-to-ASHA communication, such as through meetings, radio shows or newsletters	3
Impact	Health outcomes	ASHA programme-related changes in community healthcare-seeking, health-related behaviour and knowledge, and wellbeing/illness outcomes (including the health-related outcomes of small-scale special interventions involving ASHAs)	41
	Social outcomes	ASHA programme-related changes in non-health outcomes such as community-level environmental health and gender relations or programmatic/out-of-pocket costs (including the social-related outcomes of small-scale special interventions involving ASHAs)	6
Health services context	Health system infrastructure and functionality	The quality and availability of drugs, transportation, diagnostics, infrastructure, and health workers in the government health sector, including behaviour of health workers towards patients, and how this context influences the ASHA’s work	24
	Private sector	The quality and availability of the informal (such as traditional birth attendants) and formal private healthcare sector and how this influences the ASHA’s work, including public–private partnerships within the National Health Mission	17
	Linkages to other programmes	Intersectoral linkages between the ASHA programme (within the Ministry of Health and Family Welfare) and nutrition/*anganwadi* services through the Integrated Child Development Scheme, water and sanitation	12

In terms of CHW programme inputs, most of the articles on ASHA training and capacity-building discussed these inputs alongside other health system concerns. Only two publications took training and capacity-building as their primary focus – one commentary on training [142] and one study reporting on an intervention to improve supportive supervision [71]. After training and capacity-building, programme inputs related to supply chains, incentives and remuneration, support and monitoring, and CHW roles and role clarity were all mentioned in a large number of publications (*n* = 35–42/122). Less attention was given to CHW record keeping (*n* = 16/122).

With regards to incentives and remuneration, 12 (10%) articles focused specifically on this topic, including research on the link between incentives and performance, the influence of introducing payment vouchers for home-based newborn care [138], how adding incentives influenced the *Mitanin* programme [127] and many studies examining aspects of the *Janani Suraksha Yojana* programme, which provides ASHAs and pregnant women with cash incentives to promote institutional delivery and postnatal care [52, 53, 55, 92, 132, 133, 137]. Those that had a specific focus on the support and monitoring relationship between ASHAs and frontline health workers included two that tried pairing ASHAs with male health workers [50, 51] and one on trust and teamwork [57].

Programme outcomes, and especially ASHA performance, were discussed in more than half of the articles, and was the focus of 11 (9%) articles, including research linking ASHA education or selection with ASHA performance [65, 66, 98, 112], mHealth to improve performance [64, 74, 120, 121], the tasks ASHAs perform and the extent to which they reach marginalised households [116], their motivation to perform [109], and issues of burn out [96]. Other aspects of programme outcomes, such as CHW knowledge and motivation, also received fair attention (25%, *n* = 30/122), in contrast to CHW equity (13%, *n* = 16/122) and CHW retention and attrition (7%, *n* = 9/122).

In terms of the CHW–community interface, the most frequently discussed sub-topics were community engagement with CHWs (28%, *n* = 34/122) and resonance of CHW activities with community members (18%, *n* = 22/122). However, few articles examined community selection of CHWs (13%, *n* = 16/122), links to local government (the Panchayati Raj institutions) (12%, *n* = 15/122), the role of health committees (11%, *n* = 13/122), community oversight and support for CHWs (8%, *n* = 10/122), and CHW as a community representative (7%, *n* = 8/122). Ten articles (8%) focused specifically on the CHW–community interface, including ASHA relationships with the community [36, 40, 69, 143], village health and nutrition days [59, 99], health committees [43, 144], a maternal health video dissemination intervention [70], and ASHA communication and leadership in their communities [68].

Most sub-topics within programme governance were rarely discussed in the literature. Although the programme policy’s design and development was commented on in 27 articles, few articles discussed CHW programme oversight and guidance (*n* = 8), CHW political support (*n* = 7), the role of NGO actors in CHW policy (*n* = 6), grievance redressal for CHWs (*n* = 5), programme financing (*n* = 5), or CHW programme reporting (*n* = 2).

Within CHW social profile and agency, many articles (34%, *n* = 41/122) included content on ASHA demographics but few discussed issues related to ASHA empowerment (11%, *n* = 14/122) or communication between ASHAs (2%, *n* = 3/122). Five articles focused specifically on ASHA rights and wellbeing, including taking gender- and rights-based perspectives on remuneration and employment conditions [9, 45, 102, 108] and other ASHA struggles related to supplies, training and payment [89].

Over one-third of the articles (34%, *n* = 41/122) provided information on health outcomes linked to the programme, but few (5%, *n* = 6/122) discussed social outcomes, such as on women’s empowerment [9, 108, 126] or cost effectiveness [49, 75, 90].

In terms of health services context, the link between the health system’s functionality and the ASHA programme was discussed in 24 articles (20%), while the private sector was only discussed in 17 articles (14%). Linkages between the ASHA programme and other programmes (such as for nutrition, water or sanitation) were only discussed in 12 (10%) articles.

### Research design

The bulk of the research undertaken on the ASHA programme was descriptive (36%, *n* = 44/122), although a fairly large proportion aimed to evaluate the influence of particular factors on ASHAs (32%, *n* = 39/122). Fewer articles were exploratory (14%, *n* = 17/122) or explanatory (195, *n* = 23/122), and nine were reflective commentaries (7%, *n* = 9/122). There were no articles that were emancipatory or predictive. See Additional file 6 for a detailed classification of each article.

Among descriptive studies, the great majority, whether quantitative, qualitative or mixed, focused on the knowledge or performance of ASHAs on specific health conditions. Substantial numbers of descriptive surveys were undertaken by ASHAs to screen for health conditions, with the accuracy of such screening verified by other data sources in several studies. Outlier descriptive studies include a post-intervention survey to measure the effect of ASHAs in reducing obstetric delays [37], a mixed methods study that combined system-generated data with key informant interviews to assess health system readiness, including that of ASHAs, for malaria [54], and a formative study for an mHealth intervention inclusive of ASHAs [121].

Most influence research studies that assessed the effectiveness of ASHAs involved programme research with little consideration of health systems dimensions. Furthermore, findings on ASHAs were often subsumed among a range of frontline workers who were assessed. Outlier studies with health systems elements included a mixed methods evaluation on the impact of the *Janani Suraksha Yojana* programme on ASHA motivation and performance, among other factors [52]. Other studies sought to evaluate the effect of home-based neonatal care incentives on knowledge and practices [138], increasing knowledge of safe medical abortion on average monthly client load at health centres [118], and influence of ASHAs on immunisation coverage [145].

Among exploratory studies, two were quantitative in nature. One developed a scale to measure communication and leadership of ASHAs [68] and another developed a framework for mHealth adoption [74]. There was only one mixed methods exploratory study that sought to understand the underlying barriers to using emergency contraception among a range of providers, including ASHAs [146]. The majority of the qualitative exploratory studies sought to understand the underlying mechanisms of the ASHA programme [62, 89, 140], barriers to point-of-care testing, including by ASHAs [111], and the potential for new areas of work for ASHAs, whether cardiovascular health [125], non-communicable diseases [122], or treatment of child pneumonia [36]. Insightful health systems exploratory research included that exploring task-sharing of ASHAs with male workers [50, 51], coordination among frontline workers [109], and ethnographies by Mishra [56] and Nordfeldt and Roalkvam [40].

Explanatory mixed methods studies also discussed in more depth the underlying factors supporting overall programme performance of ASHAs [44], their role in health systems that fail women seeking obstetric care [134], mHealth interventions used by ASHAs [70, 85] and diarrhoea management including that of ASHAs [38]. Two studies focused on motivation and emotional labour, one using a Likert scale [53] and the other structural equation modelling [96]. Mixed methods explanatory studies also sought to understand questions of remuneration and the feminisation of labour [45, 102].

Amongst qualitative explanatory studies, case study research was used for an in-depth understanding of the ASHA programme [100, 126, 130]. In-depth ethnographies included those on integration and teamwork [57], notions of citizenship [108], incentives [127] and community participation [103].

Various reviews provided insight on the ASHA programme. Some drew from previous CHW experiences in India to flag issues for the current ASHA programme [147], particularly with regards to rights of ASHAs [9]. Others drew from international experience to generate lessons for how best to use CHWs for immunisation in India [148], understand CHW scale-up [149], CHW remuneration [6] or systems integration [23].

Several reviews reflected health policy and systems issues relevant to the ASHA programme, whether related to community processes [143], system readiness for newborn care [150], HIV [79], or CHW scale-up in general [151]. Some reviews were particularly critical of previous government training efforts and the implications for the ASHA programme [142] and were concerned about the overall nature of the ASHA programme [152–155].

### Authorship analysis: who is publishing on the ASHA programme?

Over half (59%, *n* = 72/122) of the papers were written by authors solely affiliated with domestic (Indian) institutions and over one-quarter (30%, *n* = 37/122) were produced by partnerships between domestic and foreign institutions. Only 11 (9%) were written by authors solely from foreign institutions. This pattern of strong domestic involvement in research either solo or in partnership with foreign organisations was further emphasised with first authorship. Almost three-quarters of the articles’ first authors are affiliated with Indian institutions (74%, *n* = 90/122), with the remaining first authors affiliated with foreign (24%, *n* = 29/122) and global (3%, *n* = 3/122) organisations. Among articles produced by partnership between domestic and foreign organisations, first authorship was 50% domestic and 50% foreign.

The location of authorship was associated with the article type. Negative findings represented a minority of the overall studies, but were largely reported by domestic first authors (20%, *n* = 18/90), as opposed to foreign first authors (7%, *n* = 2/29). In terms of research design and typology (Table 3), domestic first authors wrote all the reflective commentaries and the majority of the descriptive and explanatory research articles (89%, *n* = 39/44), while foreign first authors dominated review articles. With regards to influence studies, there was a shift from Indian first authors leading articles for simpler designs (adequacy and plausibility), with partnerships with foreign authors being more important for probability designs and exploratory studies.

**Table 3:** Domestic versus foreign authorship by research typology

Article typology	First author is:			Row total
	Domestic	Foreign	Other	
Descriptive	39	4	1	44
Explanatory	13	4	0	17
Explanatory (Review)	1	5	0	6
Exploratory	10	7	0	17
Influence: adequacy	8	2	1	11
Influence: plausibility	4	1	0	5
Influence: probability	7	5	1	13
Reflective commentary	9	0	0	9
Column total	91	28	3	122

We assessed funding sources for research on the ASHA programme and found that 49 of the 122 articles (40%) did not disclose any information on funding, while for 8 articles the authors stated that they received no funding for their work. Of those that did disclose funding, foundations (such as the Bill and Melinda Gates Foundation) were the largest funders (15%, *n* = 18/122), bilateral organisations (such as USAID) funded 10 (8%), and the Government of India funded research that was published in 9 (7%) articles.

## Discussion

### Location

As expected, research proliferated in many of the states with the highest burden of maternal and child mortality, including the larger states, such as Bihar, Jharkhand, Madhya Pradesh, Odisha, Rajasthan, Uttar Pradesh and Chhattisgarh, and the smaller north-eastern states of Arunachal Pradesh, Assam and Manipur. Two large high-burden states did not feature in the published literature – Himachal Pradesh, where the ASHA programme started later, and Jammu and Kashmir, where security concerns likely hindered research. Many of the smaller states in north-eastern India (Meghalaya, Mizoram, Nagaland, Sikkim and Tripura) also did not feature, potentially due to lower interest from research donor organisations or access barriers. Several southern states with comparatively good maternal and child health indicators were also the sites of a number of studies (particularly Andhra Pradesh, Karnataka and Kerala), often featuring research on frontier areas for ASHAs, including mental health [110], cardiovascular disease [85, 125] and other non-communicable disease screening, prevention and management [122, 123]. While several studies that supported comparative research across several states were found, additional work of this nature would support generalisations for the national programme.

### Health condition focus

Research on the routine programme tended to focus on maternal and child health, while the special interventions showcased a plethora of innovation, often emphasising special focus areas (HIV, childhood disability developmental delay, death tracking, abortion, contraceptives, uterine prolapse) that were beyond the initial purview of the ASHA programme. On the one hand, the proliferation of donor-funded pilot projects without a strong linkage to or consideration of the broader health system suggests a problematic allocation of resources to efforts unlikely to function at scale. In addition, publications that described using ASHAs merely as data collectors for other teams raise questions about coherence, permission and respecting ASHA time. On the other hand, some smaller scale studies, such as those focused on home-based newborn care, started as special interventions but were subsequently adopted into the routine programme.

### Evaluation outcomes

While smaller scale special interventions were generally able to bring about positive outcomes, research on aspects of the routine ASHA programme more often showed mixed or negative outcomes. This indicates that, with significant inputs and resources, ASHAs can deliver positive results, but that these focused short-term engagements do not reflect the everyday operating reality of the large-scale routine programme. The routine programme grapples with the challenges of operating at scale and of integration with the resource-constrained government health system.

Both positive and negative outcomes associated with ASHAs at scale occurred in the context of the National Health Mission. Research findings from specific states have shown that ASHAs have been selected across most villages [145] according to recruitment norms [112], and have received most or all of their training [44, 54]. The majority have a reasonable grasp of core health concepts [41, 46, 76, 97, 104, 131, 136], are reasonably well known and trusted as a source of health information and referral in their communities [57, 62], and are providing a subset of households with health information and services [41, 44, 56, 58, 60, 75, 101, 140], particularly by encouraging antenatal care and institutional delivery [52, 55, 132] and childhood immunisation [116]. The ASHA programme at scale has been associated with improvements in neonatal health, some aspects of care-seeking, and increased immunisation and health-related awareness in certain areas [36, 86, 135, 141, 145, 156].

Negative research results from specific states identified ASHA knowledge gaps [36, 104, 105, 114, 119, 131], inadequacies in ASHA training or supervision [56, 57, 68, 93, 105, 109, 139, 146, 156], low community engagement with and awareness of ASHAs [134], challenges related to referrals (limited transportation, coordination and health facility resources) [55, 100, 111, 136, 140], dissatisfaction among ASHAs with their remuneration or support [9, 52, 53, 89, 102, 140], lack of supplies [54, 60, 89, 156], and subpar performance or coverage [41, 60, 65, 66, 75, 103, 112, 116, 145].

Chhattisgarh’s *Mitanin* programme emerged as a strong success story, wherein *Mitanins* performed as socio-political actors on the social determinants of health [126, 128, 130]. However, in other states, ASHAs have generally been more successful in performing a link-worker role, without significant action on community mobilisation or the social determinants of health [43, 44, 98, 100, 152].

### Health systems perspective

The National Health Mission is a health system reform seeking to strengthen government service delivery through all the health system building blocks, including cross-cutting community engagement. However, health condition-focused research on the ASHA programme often took a narrow perspective, without accounting for the broader health system factors in which the programme operates. In comparison, studies on general ASHA work examined the programme in context and considered broader systemic or cross-cutting policy issues.

### Health systems content

Many publications presented meaningful information on multiple topics in the CHW–health system framework (Fig. 1), reflecting that researchers frequently considered the ASHA programme within the broader health system context. However, a number of essential health system considerations, which are central to the programme’s vision, were rarely discussed in the published literature. In particular, there was little consideration of programme governance (programme oversight and guidance, CHW political support, the role of NGO actors in CHW policy, grievance redressal for CHWs, programme financing, and CHW programme reporting), community voice, community engagement in ASHA selection, and community collaboration with ASHAs through health committees. While many studies measured ASHA performance and knowledge, few discussed or used existing programme or health facility records, leaving the routine systems needed to monitor the programme unexplored. Additionally, while health outcomes associated with the ASHA programme or small-scale interventions engaging ASHAs were frequently assessed, social outcomes were rarely discussed. While the existing research on gender, motivation, ASHA agency, and relationships and linkages among different actors (auxiliary nurse midwives, *Anganwadi* workers, block and district managers) is welcome, far more research on these topics is required to truly understand the ASHA as an integrated member of the health system.

In the first decade of the programme, research assessing ASHA capacity and performance has played a valuable role in understanding early challenges and successes. However, as the ASHA programme enters its next decade, research on other aspects of the CHW–health system framework will be increasingly important to the programme’s capacity to adapt, sustain and achieve its broader goals around empowerment, community engagement and change across the social determinants of health. Future research should consider the upcoming challenges of running a mature CHW programme at scale, including recruitment and training for expanded roles in non-communicable diseases, ASHA social security, retention, aging, and ongoing knowledge retention and skills upgrade. Furthermore, echoing global gaps in research on CHW programmes [13], ongoing research is required on meeting the rights and needs of ASHAs, effective approaches to training and supervision, on realising the ASHA role as a community change agent, and on the influence of health system decentralisation, social accountability and governance.

### Research typology and methodology

The academic literature on the ASHA programme showcases a rich range of research. It is noteworthy that research focusing directly on aspects of the ASHA programme itself tended to be descriptive and explanatory, while the more complex influence studies, including the randomised controlled trials, focused on broader health interventions, such as integrated management of childhood illness, with limited findings pertaining to ASHAs, who were just one of many providers.

The plethora of descriptive work offered important early feedback into the programme’s condition, including the extent to which it was implemented as intended and key issues of ASHA capacity and performance. However, as the programme moves forward into its next decade, policy-makers would benefit from stronger evidence on the programme’s impact on health and social outcomes, as well as on the optimal mechanisms for ASHA training, support and remuneration. Rigorous explanatory studies and health systems evaluations, such as mixed methods influence studies and realist evaluations, taking these areas of the ASHA programme as their focus, would enable increasingly evidence-informed policy-making, planning and advocacy. Furthermore, there is a need for emancipatory research that engages ASHAs as agents to identify and work towards addressing challenges, and predictive research that helps guide next steps. Ultimately, a complex, evolving and dynamic intervention such as the ASHA programme, which is implemented at scale as part of a larger system reform agenda, cannot be studied through a single research design. Research agendas at state and national levels are likely to be different and the various facets of the programme require different designs.

### Authorship and funding

Indian authors and institutions have shown strong research leadership and critical engagement with the programme, and have also developed international partnerships to support complex research studies. The dominance of Indian authors, especially in critiques of the programme and research with negative and mixed findings, showcases a vibrant civil society space – and is a welcome contrast to other country contexts where critical research may be severely curtailed [157]. While the dominance of Indian-led academic work generates opportunities to disseminate and build upon the research findings in the same setting [158], there was repetition among descriptive studies and other areas of inquiry were largely neglected. Further engagement is needed between policy needs and research generation to ensure effective use of research investments.

The high percentage of papers that did not disclose their source of funding is concerning, as it could obscure power dynamics guiding research priority topics, regions and institutions. It is also noteworthy that research commissioned and funded by the government was largely absent from the published literature, although we note that this research may be conducted and disseminated internally or published in the grey literature. A mature CHW programme requires government leadership in funding research that addresses policy-maker needs, including large scale evaluations. Ensuring that this research is peer reviewed and published in journals will help to inform national and international conversations on CHWs.

### Limitations

This overview of published literature on the ASHA programme has several limitations. We only considered published studies listed in prominent journal databases (PubMed, Scopus and Embase). We did not include the extensive grey literature published on the programme, including important NGO and government reports. While we also gathered and analysed all government documents in the public domain, the findings from that area of work are reported on elsewhere [11] and are beyond the scope of this paper.

Authorship, while classified as domestic, foreign and other, hides those of Indian origin based in foreign institutions, or who may have subsequently moved back to India. Some included articles significantly involved ASHAs in their studies but had limited findings relevant to the ASHA programme (e.g. studies that used ASHAs as data collectors to assess the prevalence of a health issue, but were not ultimately assessing or commenting on the ASHA role or capacity). Other studies did not disaggregate ASHAs from other frontline health workers.

We did not assess the methodological quality of the included articles because there was such a wide range of research typologies used. This limits our ability to comment on the strength of research being done and on how trustworthy, robust, and meaningful these academic contributions are. The wide range in complexity, insight, and rigor across the included manuscripts is not analysed in this paper.

## Conclusion

Academic work on the ASHA programme highlights a range of special interventions, but also showcases the challenges faced by a programme at massive scale, situated in the broader health system. As the programme continues to evolve, ongoing research and continued domestic critical leadership is vital to address key knowledge gaps and provide insight into ground realities, including on programme governance, intersectoral linkages, ASHA solidarity, and community capacity to provide support and oversight.

## Additional files

Additional file 1: Search terms on the ASHA community health worker programme. (DOCX 18 kb)

Additional file 2: Reference list of all included articles. (DOCX 31 kb)

Additional file 3: Database of included articles and their characteristics. (XLSX 73 kb)

Additional file 4: Summary of research on the main ASHA programme. (DOCX 71 kb)

Additional file 5: Summary of smaller scale interventions that engaged ASHAs. (DOCX 44 kb)

Additional file 6: Research on ASHAs, by typology and methodology. (DOCX 133 kb)

## Abbreviations

ASHA: Accredited social health activist
CHW: Community health worker
HIV: Human immunodeficiency virus

## Footnotes

1 Chandigarh, Dadra and Nagar Haveli, Daman and Diu, Lakshadweep, Puducherry, Andaman and Nicobar Islands.
