# Supplementary material for: Taking stock of 10 years of published research on the ASHA programme: examining India’s national community health worker programme from a health systems perspective
Source: Health Res Policy Syst. 2019 Mar 25;17:29. doi: 10.1186/s12961-019-0427-0 (PMC6434894; doi:10.1186/s12961-019-0427-0)
Supplement: Supplementary file 1 — Search terms on the ASHA community health worker programme. (DOCX 18 kb) [file 12961_2019_427_MOESM1_ESM.docx]

Additional file 1. Search terms on the ASHA community health worker program

Pubmed

Concept 1. Community health worker

}mitanin} OR }Mitanin} OR }sahyogini} OR }Sahyogini} OR }ASHA} or }Accredited Social Health Activist} OR }accredited social health activist} OR }health auxiliary} OR }frontline health workers} OR }frontline health worker} OR }outreach worker} OR }outreach workers} OR }lay health worker} OR }lay health workers} OR }village health worker} OR }village health workers} OR }volunteer health worker} OR }volunteer health workers} OR }voluntary health workers} OR }voluntary health worker} OR }community health agent} OR }community health agents} OR }health promoter} OR }health promoters} OR }Community Health Workers}[Mesh] OR }community health worker} OR }community health workers} OR }community health aide} OR }community health aides} OR }community health volunteer} OR }community health volunteers} OR }community health assistants} OR }community health assistant} OR }community health promoters} OR }community health promoters} OR }community volunteer} OR }community volunteers} OR }health extension workers} OR }health extension worker} OR }village health volunteer} OR }village health volunteers} OR }Community Health Nursing}[Mesh] OR }close-to-community provider} OR }close-to-community providers} OR }community-based practitioner} OR }community-based practitioners} OR }Community Practitioners} OR }Community Practitioner} OR }community-based practitioners} OR }community-based practitioner} OR }rural health auxiliaries} OR }Basic health worker} OR }Basic health workers} OR }Community health agent} OR }Community health agents} OR }Community health promoter} OR }Community health promoters} OR }Community health representative} OR }Community health representatives} OR }Community health volunteer} OR }Community health volunteers} OR }Community resource person} OR }Female multipurpose health worker} OR }Female multipurpose health worker} OR }Health promoter} OR }Health promoters} OR }Outreach educator} OR }Outreach educators} OR }Sevika} OR }Village health helper} OR }Community Case Management Workers} OR }Community Health Agent} OR }Community Health Agents} OR }Community Health Care Provider} OR }Community Health Care Providers} OR }Community HealthCare Provider} OR }Community HealthCare Providers} OR }Community Health Extension Worker} OR }Community Health Extension Workers} OR }Family Health Worker} OR }Family Health Workers} OR }Family Planning Agent} OR }Family Planning Agents} OR }Family Welfare Assistant} OR }Family Welfare Assistants} OR }Female Community Health Volunteer} OR }Female Community Health Volunteers} OR }Health Agent} OR }Health Agents} OR }Health Assistant} OR }Health Assistants} OR }Maternal and Child Health Worker} OR }Maternal and Child Health Workers} OR }Peer Educator} OR }Peer Educators}

Concept 2. Location: India

India[mesh] OR Indian[tiab] OR }Andhra Pradesh} [tiab] OR }Arunachal Pradesh} [tiab] OR Assam[tiab] OR Bihar[tiab] OR Chhattisgarh[tiab] OR Goa[tiab] OR Gujarat[tiab] OR Haryana[tiab] OR }Himachal Pradesh} [tiab] OR Jammu[tiab] OR Kashmir[tiab] OR Jharkhand[tiab] OR Karnataka[tiab] OR Kerala[tiab] OR }Madhya Pradesh} [tiab] OR Maharashtra[tiab] OR Manipur[tiab] OR Meghalaya[tiab] OR Mizoram[tiab] OR Nagaland[tiab] OR Odisha[tiab] OR Orissa[tiab] OR Punjab[tiab] OR Rajasthan[tiab] OR Sikkim[tiab] OR }Tamil Nadu} [tiab] OR Telangana[tiab] OR Tripura[tiab] OR }Uttar Pradesh} [tiab] OR Uttarakhand[tiab] OR }West Bengal} [tiab] OR Andaman[tiab] OR Nicobar[tiab] OR Chandigarh[tiab] OR Dadra[tiab] OR }Nagar Haveli} [tiab] OR Daman[tiab] OR Diu[tiab] OR Lakshadweep[tiab] OR Delhi[tiab] OR Puducherry[tiab] OR Pondicherry[tiab]

EMBASE

Concept 1. Community health worker

}mitanin} OR }Mitanin} OR }sahyogini} OR }Sahyogini} OR }ASHA} or }Accredited Social Health Activist} OR }accredited social health activist} OR 'health auxiliary'/exp OR }health auxiliary} OR }frontline health workers} OR }frontline health worker} OR }outreach worker} OR }outreach workers} OR }lay health worker} OR }lay health workers} OR }village health worker} OR }village health workers} OR }volunteer health worker} OR }volunteer health workers} OR }voluntary health workers} OR }voluntary health worker} OR }community health agent} OR }community health agents} OR }health promoter} OR }health promoters} OR }Community Health Workers} OR }community health worker} OR }community health workers} OR }community health aide} OR }community health aides} OR }community health volunteer} OR }community health volunteers} OR }community health assistants} OR }community health assistant} OR }community health promoters} OR }community health promoters} OR }community volunteer} OR }community volunteers} OR }health extension workers} OR }health extension worker} OR }village health volunteer} OR }village health volunteers} OR }Community Health Nursing} OR }Community Health Nursing}/exp OR }close-to-community provider} OR }close-to-community providers} OR }community-based practitioner} OR }community-based practitioners} OR }Community Practitioners} OR }Community Practitioner} OR }community-based practitioners} OR }community-based practitioner} OR }rural health auxiliaries} OR }Basic health worker} OR }Basic health workers} OR }Community health agent} OR }Community health agents} OR }Community health promoter} OR }Community health promoters} OR }Community health representative} OR }Community health representatives} OR }Community health volunteer} OR }Community health volunteers} OR }Community resource person} OR }Female multipurpose health worker} OR }Female multipurpose health worker} OR }Health promoter} OR }Health promoters} OR }Outreach educator} OR }Outreach educators} OR }Sevika} OR }Village health helper} OR }Community Case Management Workers} OR }Community Health Agent} OR }Community Health Agents} OR }Community Health Care Provider} OR }Community Health Care Providers} OR }Community HealthCare Provider} OR }Community HealthCare Providers} OR }Community Health Extension Worker} OR }Community Health Extension Workers} OR }Family Health Worker} OR }Family Health Workers} OR }Family Planning Agent} OR }Family Planning Agents} OR }Family Welfare Assistant} OR }Family Welfare Assistants} OR }Female Community Health Volunteer} OR }Female Community Health Volunteers} OR }Health Agent} OR }Health Agents} OR }Health Assistant} OR }Health Assistants} OR }Maternal and Child Health Worker} OR }Maternal and Child Health Workers} OR }Peer Educator} OR }Peer Educators}

Concept 2. Location: India

India/exp OR (India OR Indian OR }Andhra Pradesh} OR }Arunachal Pradesh} OR Assam OR Bihar OR Chhattisgarh OR Goa OR Gujarat OR Haryana OR }Himachal Pradesh} OR Jammu OR Kashmir OR Jharkhand OR Karnataka OR Kerala OR }Madhya Pradesh} OR Maharashtra OR Manipur OR Meghalaya OR Mizoram OR Nagaland OR Odisha OR Orissa OR Punjab OR Rajasthan OR Sikkim OR }Tamil Nadu} OR Telangana OR Tripura OR }Uttar Pradesh} OR Uttarakhand OR }West Bengal} OR Andaman OR Nicobar OR Chandigarh OR Dadra OR }Nagar Haveli} OR Daman OR Diu OR Lakshadweep OR Delhi OR Puducherry OR Pondicherry):ab,ti

Scopus

Concept 1. Community health worker

{mitanin} OR {Mitanin} OR {sahyogini} OR {Sahyogini} OR {ASHA} or {Accredited Social Health Activist} OR {accredited social health activist} OR 'health auxiliary'/exp OR {health auxiliary} OR {frontline health workers} OR {frontline health worker} OR {outreach worker} OR {outreach workers} OR {lay health worker} OR {lay health workers} OR {village health worker} OR {village health workers} OR {volunteer health worker} OR {volunteer health workers} OR {voluntary health workers} OR {voluntary health worker} OR {community health agent} OR {community health agents} OR {health promoter} OR {health promoters} OR {Community Health Workers} OR {community health worker} OR {community health workers} OR {community health aide} OR {community health aides} OR {community health volunteer} OR {community health volunteers} OR {community health assistants} OR {community health assistant} OR {community health promoters} OR {community health promoters} OR {community volunteer} OR {community volunteers} OR {health extension workers} OR {health extension worker} OR {village health volunteer} OR {village health volunteers} OR {Community Health Nursing} OR {Community Health Nursing}/exp OR {close-to-community provider} OR {close-to-community providers} OR {community-based practitioner} OR {community-based practitioners} OR {Community Practitioners} OR {Community Practitioner} OR {community-based practitioners} OR {community-based practitioner} OR {rural health auxiliaries} OR {Basic health worker} OR {Basic health workers} OR {Community health agent} OR {Community health agents} OR {Community health promoter} OR {Community health promoters} OR {Community health representative} OR {Community health representatives} OR {Community health volunteer} OR {Community health volunteers} OR {Community resource person} OR {Female multipurpose health worker} OR {Female multipurpose health worker} OR {Health promoter} OR {Health promoters} OR {Outreach educator} OR {Outreach educators} OR {Sevika} OR {Village health helper} OR {Community Case Management Workers} OR {Community Health Agent} OR {Community Health Agents} OR {Community Health Care Provider} OR {Community Health Care Providers} OR {Community HealthCare Provider} OR {Community HealthCare Providers} OR {Community Health Extension Worker} OR {Community Health Extension Workers} OR {Family Health Worker} OR {Family Health Workers} OR {Family Planning Agent} OR {Family Planning Agents} OR {Family Welfare Assistant} OR {Family Welfare Assistants} OR {Female Community Health Volunteer} OR {Female Community Health Volunteers} OR {Health Agent} OR {Health Agents} OR {Health Assistant} OR {Health Assistants} OR {Maternal and Child Health Worker} OR {Maternal and Child Health Workers} OR {Peer Educator} OR {Peer Educators}

Concept 2. Location: India

TITLE-ABS({India} OR {Indian} OR {Andhra Pradesh} OR {Arunachal Pradesh} OR {Assam} OR {Bihar} OR {Chhattisgarh} OR {Goa} OR {Gujarat} OR {Haryana} OR {Himachal Pradesh} OR {Jammu} OR {Kashmir} OR {Jharkhand} OR {Karnataka} OR {Kerala} OR {Madhya Pradesh} OR {Maharashtra} OR {Manipur} OR {Meghalaya} OR {Mizoram} OR {Nagaland} OR {Odisha} OR {Orissa} OR {Punjab} OR {Rajasthan} OR {Sikkim} OR {Tamil Nadu} OR {Telangana} OR {Tripura} OR {Uttar Pradesh} OR {Uttarakhand} OR {West Bengal} OR {Andaman} OR {Nicobar} OR {Chandigarh} OR {Dadra} OR {Nagar Haveli} OR {Daman} OR {Diu} OR {Lakshadweep} OR {Delhi} OR {Puducherry} OR {Pondicherry})
