# Supplementary material for: Taking stock of 10 years of published research on the ASHA programme: examining India’s national community health worker programme from a health systems perspective
Source: Health Res Policy Syst. 2019 Mar 25;17:29. doi: 10.1186/s12961-019-0427-0 (PMC6434894; doi:10.1186/s12961-019-0427-0)
Supplement: Supplementary file 2 — Reference list of all included articles. (DOCX 31 kb) [file 12961_2019_427_MOESM2_ESM.docx]

**Additional file 2: Included articles (listed in the order that the articles are presented in additional file 6: Research on ASHAs, by typology and methodology)**

1. Fathima FN, Raju M, Varadharajan KS, Krishnamurthy A, Ananthkumar SR, Mony PK. Assessment of “Accredited Social Health Activists” -- A National Community Health Volunteer Scheme in Karnataka State, India. J Heal Popul Nutr. 2015;33(1):137–45.

2. Ramadurg U, Vidler M, Charanthimath U, Katageri G, Bellad M, Mallapur A, et al. Community health worker knowledge and management of pre-eclampsia in rural Karnataka State, India. Reprod Health [Internet]. 2016;13(Suppl 2):113. Available from: http://www.ncbi.nlm.nih.gov/pubmed/27719678

3. Sinha LN, Kaur P, Gupta R, Dalpath S, Goyal V, Murhekar M. Newborn care practices and home-based postnatal newborn care programme - Mewat, Haryana, India, 2013. West Pacific Surveill response J WPSAR [Internet]. 2014;5(3):Russmann, W., Konig, U., Schlimbach, K., Pawlowska. Available from: http://www.scopus.com/inward/record.url?eid=2-s2.0-84965191350&partnerID=tZOtx3y1

4. Srivastava D, Prakash S, Ashish V, Nair K, Gupta S, Nandan D. A Study of Interface of ASHA with the Community and the Service Providers in Eastern Uttar Pradesh. Indian J Public Health. 2009;53(3):133.

5. Thacker N, Choudhury P, Gargano LM, Weiss PS, Pazol K, Vashishtha VM, et al. Attitudes and practices of auxiliary nurse midwives and accredited social health activists in uttar pradesh and bihar regarding polio immunization in india. J Trop Pediatr. 2013;59(4):266–73.

6. Deshpande S, Bhanot A, Maknikar S. Assessing the Influence of a 360-degree Marketing Communications Campaign With 360-degree Feedback. Soc Mar Q [Internet]. 2015;21(3):142–51. Available from: http://smq.sagepub.com/lookup/doi/10.1177/1524500415599528

7. Hussain M a, Dandona L, Schellenberg D. Public health system readiness to treat malaria in Odisha State of India. Malar J [Internet]. 2013;12(1):351. Available from: http://www.pubmedcentral.nih.gov/articlerender.fcgi?artid=3850888&tool=pmcentrez&rendertype=abstract

8. Panigrahi S, Mohapatra B, Mishra K. Awareness, perception and practice of stakeholders in India regarding Village Health and Nutrition Day. J Fam Med Prim Care. 2015;4(2):244.

9. Saxena V, Kumari R, Kumar P, Nath B, Pal R. Planning and preparation of VHND through convergence: Sharing experiences from Uttarakhand. Clin Epidemiol Glob Heal [Internet]. 2015;3(3):125–31. Available from: http://dx.doi.org/10.1016/j.cegh.2014.11.001

10. Sundararaman T, Ved R, Gupta G, Samatha M. Determinants of functionality and effectiveness of community health workers: results from evaluation of ASHA program in eight Indian states. BMC Proc [Internet]. 2012;6(Suppl 5):O30. Available from: http://www.biomedcentral.com/1753-6561/6/S5/O30

11. Mony P, Raju M. Evaluation of ASHA programme in Karnataka under the National Rural Health Mission. BMC Proc [Internet]. 2012;6(Suppl 5):P12. Available from: http://www.biomedcentral.com/1753-6561/6/S5/P12

12. Malini S, Tripathi RM, Khattar P, Nair KS, Tekhre YL, Dhar N, et al. A Rapid Appraisal on Fuctioning of janani Suraksha Yojana in South Orrisa. Heal Poluplation. 2008;31(2):126–31.

13. Modi D, Gopalan R, Shah S, Venkatraman S, Desai G, Desai S, et al. Development and formative evaluation of an innovative mHealth intervention for improving coverage of community-based maternal, newborn and child health services in rural areas of India. Glob Health Action. 2015;8(1).

14. Bhargavi CN, Sharma A. MCH services in Delhi in terms of beneficiaries’ awareness, coverage and satisfaction. Nurs J India. 2014;105(4):186–90.

15. Kohli C, Kishore J, Sharma S, Nayak H. Knowledge and practice of Accredited Social Health Activists for maternal healthcare delivery in Delhi. J Fam Med Prim care [Internet]. 2015;4(3):359–63. Available from: http://www.ncbi.nlm.nih.gov/pubmed/26288774

16. S. K, M. B. A cross-sectional assessment of knowledge of ASHA workers. J Krishna Inst Med Sci Univ [Internet]. 2015;4(4):57–63. Available from: http://www.jkimsu.com/jkimsu-vol4no4/JKIMSU, Vol. 4, No. 4, Oct-Dec 2015 Page 57-63.pdf%5Cnhttp://ovidsp.ovid.com/ovidweb.cgi?T=JS&PAGE=reference&D=emed13&NEWS=N&AN=2015482227

17. Kosec K, Avula R, Holtemeyer B, Tyagi P, Hausladen S, Menon P. Predictors of Essential Health and Nutrition Service Delivery in Bihar, India: Results From Household and Frontline Worker Surveys. Glob Heal Sci Pract [Internet]. 2015;3(2):255–73. Available from: http://www.ghspjournal.org/content/3/2/255.full

18. Kansal S, Kumar S, Kumar A. Is educational level of ASHA matters for their effective functioning? A cross- sectional study in eastern uttar pradesh. Indian J Community Heal. 2012;24(1):41–4.

19. Kumar S, Kaushik A, Kansal S. Factors Influencing the Work Performance of Asha Under Nrhm a Cross Sectional Study From Eastern Uttar Pradesh. [Internet]. Vol. 24, Indian Journal of Community Health. 2012. p. 325–31. Available from: http://iapsmupuk.org/journal/index.php/IJCH/article/view/373

20. Saxena V, Kakkar R, Semwal VD. A study on ASHA -a change agent of the society. Indian J Community Heal. 2012;24(1):15–8.

21. Bansal SC, Nimbalkar SM, Shah NA, Shrivastav RS, Phatak AG. Evaluation of knowledge and skills of home based newborn care among Accredited Social Health Activists (ASHA). Indian Pediatr. 2016;53(8):689–91.

22. Shrivastava SR, Shrivastava PS. Evaluation of trained Accredited Social Health Activist (ASHA) workers regarding their knowledge, attitude and practices about child health. Rural Remote Health. 2012;12(4):1–7.

23. Saxena V, Kumari R. Infant and young child feeding - knowledge and practices of ASHA workers of Doiwala block, Dehradun District. Indian J Community Heal [Internet]. 2014;26(1):68–75. Available from: http://www.iapsmupuk.org/journal/index.php/IJCH/article/view/565/html_13

24. Lamberti LM, Walker CLF, Taneja S, Mazumder S, Black RE. The association between provider practice and knowledge of ORS and zinc supplementation for the treatment of childhood diarrhea in Bihar, Gujarat and Uttar Pradesh, India: A multi-site cross-sectional study. PLoS One. 2015;10(6):1–17.

25. Shukla A, Bhatnagar T. Accredited Social Health Activists and pregnancy related services in Uttarakhand, India. BMC Proc [Internet]. 2012;6(Suppl 1):1–2. Available from: http://search.ebscohost.com/login.aspx?direct=true&db=aph&AN=71341375&site=ehost-live

26. Sidney K, Diwan V, El-Khatib Z, de Costa A. India’s JSY cash transfer program for maternal health: Who participates and who doesn’t - a report from Ujjain district. Reprod Health [Internet]. 2012;9(1):2. Available from: http://www.reproductive-health-journal.com/content/9/1/2

27. Vikram K, Sharma AK, Kannan AT. Beneficiary level factors influencing Janani Suraksha Yojana utilization in urban slum population of trans-Yamuna area of Delhi. Indian J Med Res. 2013;138(SEP):340–6.

28. Malaviya P, Hasker E, Singh RP, Van Geertruyden JP, Boelaert M, Sundar S. Village health workers in Bihar, India: An untapped resource in the struggle against kala-azar. Trop Med Int Heal. 2013;18(2):188–93.

29. Persai D, Panda R, Mathur MR. Self-reported Practices and Attitudes of Community Health Workers (Accredited Social Health Activist) in Tobacco Control - Findings from two states in India. Int J Prev Med. 2015;6:48.

30. Prasad H. Evaluation of malaria control programme in three selected districts of Assam, India. J Vector Borne Dis. 2009;46(4):280–7.

31. Sahu SS, Rao SP, Dash S. Performance of Accredited Social Health Activists (ASHAs) in diagnosis and treatment of Malaria in Eight Falciparum Endemic Tribal Districts of Southern Odisha, India. J Commun Dis. 2016;48(2):12–9.

32. Sagare SM, Bogam RR, Murarkarsujata SK, Patil UP, Ghate MM. Knowledge, attitude and practices of ASHAs regarding tuberculosis and DOTS. Indian J Sci Technol. 2012;5(3):2401–4.

33. Salve HR, Babu S, Rai SK, Sagar R, Kant S. Attitude about mental illness of health care providers and community leaders in rural. 2015;26(4):2015.

34. Tiwari SN, Pandey SC, Chandra U. Disability control by self-care at Home in Bihar. Indian J Lepr. 2012;84:108.

35. Verma C, Rao P. Determinants of rural women’s participation in india’s National Leprosy Eradication Programme. Indian J Lepr. 2014;86:105–10.

36. Das E, Panwar DS, Fischer EA, Bora G, Carlough MC. Performance of accredited social health activists to provide home-based newborn care: A situational analysis. Indian Pediatr. 2014;51(2):142–4.

37. Johnston HB, Ganatra B, Nguyen MH, Habib N, Afework MF, Harries J, et al. Accuracy of assessment of eligibility for early medical abortion by community health workers in Ethiopia, India and South Africa. PLoS One. 2016;11(1):1–11.

38. Bijalwan RP, Bhagavatula M, Semwal VD, Rawat P, Anand V. Morbidity of Uterine Prolapsed among the Women in the Chakrata Block of Dehradun District. Indian J Community Heal [Internet]. 2015;27(1):103–9. Available from: http://search.ebscohost.com/login.aspx?direct=true&db=a9h&AN=115317042&site=ehost-live

39. Jonnalagada S, Rao K, Chary R, M S, Rao R. A focused approach to detect leprosy in high burden tribal mandals of Adilabad district, Andhra Pradesh, India. 2012;

40. Menon J, Joseph J, Thachil A, Attacheril T V., Banerjee A. Surveillance of noncommunicable diseases by community health workers in Kerala: The Epidemiology of Noncommunicable Diseases in Rural Areas (ENDIRA) study. Glob Heart [Internet]. 2014;9(4):409–17. Available from: http://dx.doi.org/10.1016/j.gheart.2014.07.003

41. Nair MKC, Princly P, Leena ML, Swapna S, Kumari I L, Preethi R, et al. CDC Kerala 17: Early Detection of Developmental Delay / Disability Among Children Below 3??y in Kerala - A Cross Sectional Survey. Indian J Pediatr. 2014;81(2):156–60.

42. Raju M. Validating Infant and Maternal Mortality Reporting in Doddaballapur Taluk of Bangalore Rural District – a Pilot Study. From 2nd Natl Conf Bringing Evid into Public Heal Policy (EPHP 2012) [Internet]. 2012;6(Suppl 5):5–6. Available from: www.biomedcentral.com/1753-6561/6/S5/O23

43. Mishra A, Kar K, Satapathy D. Mass drug administration against filariasis - a study on coverage and compliance, in a coastal district of Odisha. J Commun Dis [Internet]. 2015;47(4):13–8. Available from: http://ismocd.org/jcd/47_4/13-18-----541-1990-1-RV.pdf

44. Modi D, Patel J, Desai S, Shah P. Accessing completeness of pregnancy, delivery, and death registration by Accredited Social Health Activists [ASHA] in an innovative mHealth project in the tribal areas of Gujarat: A cross-sectional study. J Postgrad Med [Internet]. 2016;62(3):170–2. Available from: http://10.0.16.7/0022-3859.183168%5Cnhttp://search.ebscohost.com/login.aspx?direct=true&db=a9h&AN=117029131&site=ehost-live

45. Gopalan SS, Durairaj V. Addressing maternal healthcare through demand side financial incentives: experience of Janani Suraksha Yojana program in India. BMC Health Serv Res [Internet]. 2012;12(1):319. Available from: http://www.scopus.com/inward/record.url?eid=2-s2.0-84867562946&partnerID=tZOtx3y1

46. Gogoi A, Parmar S, Katoch M, Ziauddin M. Addressing the reproductive health needs and rights of married adolescent couples. 14th Congr Eur Soc Contracept Reprod Heal. 2016;

47. Goel S, Gupta P, Aggarwal AK, Patro BK, Kaur J, Aggarwal N. Evaluation of Nischay scheme in improving antenatal care in a northern state of India. Arch Gynecol Obstet. 2013;288(4):815–9.

48. Armstrong G, Kermode M, Raja S, Suja S, Chandra P, Jorm A. A mental health training program for community health workers in India: impact on knowledge and attitudes. Int J Ment Health Syst [Internet]. 2011;5(17):1–11. Available from: http://search.ebscohost.com/login.aspx?direct=true&db=aph&AN=66601891&site=ehost-live%5Cnhttp://download.springer.com/static/pdf/517/art%253A10.1186%252F1752-4458-5-17.pdf?originUrl=http%3A%2F%2Fhttp%3A%2F%2Fijmhs.biomedcentral.com%2Farticle%2F10.1186%2F1

49. Thakre SS, Thakre SB, Thakre AD, Golawar SH, More SM, Humne AY. Effectiveness of the Training Course of ASHA on Infant Feeding Practices at a Rural Teaching Hospital: A Cross Sectional Study. J Clin Diagnostic Res [Internet]. 2012;6(6):1038–40. Available from: http://search.ebscohost.com/login.aspx?direct=true&db=aph&AN=83173160&site=ehost-live

50. Singh N. To evaluate the role of training session on ‘Cervical Cancer Screening’ in improving knowledge and attitude of Accredited Social Health Activists (ASHA) in North Indian population. Ann Oncol. 2015;26((Supplement 9)):ix80–ix84.

51. Stalin P, Krishnan A, Rai SK, Agarwal RK. ASHA’s involvement in newborn care: A feasibility study. Indian Pediatr. 2011;48(11):897–9.

52. Mahanta TG, Islam S, Sudke AK, Kumari V, Gogoi P, Rane T, et al. Effectiveness of introducing home-based newborn care (HBNC) voucher system in Golaghat District of Assam. Clin Epidemiol Glob Heal [Internet]. 2016;4(2):69–75. Available from: http://www.sciencedirect.com/science/article/pii/S2213398415000470

53. Panwar DS, Naidu V, Das E, Verma S, Khan AA. Strengthening support mechanisms for Accredited Social Health Activists in order to improve home-based newborn care in Uttar Pradesh, India. BMC Proc [Internet]. 2012;6(Suppl 5):1–2. Available from: http://search.ebscohost.com/login.aspx?direct=true&db=aph&AN=82432034&site=ehost-live

54. Singh R. Community outreach to improve medical abortion access in India. Int J Gynecol Obstet [Internet]. 2009;107:S77. Available from: http://www.sciencedirect.com/science/article/pii/S002072920960309X

55. Wagner AL, Bettampadi D, Porth JM, Boulton ML. The impact of increased use of ASHAs on rural immunization coverage in India. 17th Int Congr Infect Dis [Poster] [Internet]. 2016;45:209–10. Available from: http://dx.doi.org/10.1016/j.ijid.2016.02.478

56. Mohan P, Kishore B, Singh S, Bahl R, Puri A, Kumar R. Assessment of implementation of integrated management of neonatal and childhood illness in India. J Heal Popul Nutr [Internet]. 2011;29(6):629–38. Available from: http://www.ncbi.nlm.nih.gov/pubmed/22283037

57. Das VNR, Pandey RN, Kumar V, Pandey K, Siddiqui NA, Verma RB, et al. Repeated training of accredited social health activists (ASHAs) for improved detection of visceral leishmaniasis cases in Bihar, India. Pathog Glob Health [Internet]. 2016;110(1):33–5. Available from: http://www.tandfonline.com/doi/full/10.1080/20477724.2016.1156902

58. Padda P, Devgun S, Gupta V, Chaudhari S, Singh G. Role of ASHA in improvement of maternal health status in northern India: An urban rural comparison. Indian J Community Heal. 2013;25(4):465–71.

59. Valadez JJ, Devkota B, Pradhan MM, Meherda P, Sonal GS, Dhariwal A, et al. Improving malaria treatment and prevention in India by aiding district managers to manage their programmes with local information: a trial assessing the impact of Lot Quality Assurance Sampling on programme outcomes. Trop Med Int Health. 2014;19(10):1226–36.

60. Vir SC, Kalita A, Mondal S, Malik R. Impact of community-based mitanin programme on undernutrition in rural Chhattisgarh State, India. Food Nutr Bull. 2014;35(1):83–91.

61. DeRenzi B, Wacksman J, Dell N, Lee S, Lesh N, Borriello G, et al. Closing the Feedback Loop: A 12-month Evaluation of ASTA, a Self-Tracking Application for ASHAs. Proc Eighth Int Conf Inf Commun Technol Dev [Internet]. 2016;22:1--22:10. Available from: http://doi.acm.org/10.1145/2909609.2909652

62. Tripathy P, Nair N, Sinha R, Rath S, Gope RK, Rath S, et al. Effect of participatory women’s groups facilitated by Accredited Social Health Activists on birth outcomes in rural eastern India: A cluster-randomised controlled trial. Lancet Glob Heal [Internet]. 2016;4(2):e119–28. Available from: http://dx.doi.org/10.1016/S2214-109X(15)00287-9

63. Prinja S, Mazumder S, Taneja S, Bahuguna P, Bhandari N, Mohan P, et al. Cost of delivering child health care through community level health workers: How much extra does IMNCI program cost? J Trop Pediatr. 2013;59(6):489–95.

64. Bhandari GP, Subedi N, Thapa J, Choulagai B, Maskey MK, Onta SR. A cluster randomized implementation trial to measure the effectiveness of an intervention package aiming to increase the utilization of skilled birth attendants by women for childbirth: Study protocol. BMC Pregnancy Childbirth [Internet]. 2014;14(1). Available from: https://www.scopus.com/inward/record.uri?eid=2-s2.0-84899112724&doi=10.1186%2F1471-2393-14-109&partnerID=40&md5=37dda4f50019a872e3937f413438b599

65. Mazumder S, Taneja S, Bahl R, Mohan P, Strand TA, Sommerfelt H, et al. Effect of implementation of integrated management of neonatal and childhood illness programme on treatment seeking practices for morbidities in infants: cluster randomised trial. BMJ [Internet]. 2014;349(August):g4988–g4988. Available from: http://search.ebscohost.com/login.aspx?direct=true&db=cmedm&AN=25172514&site=ehost-live%5Cnhttp://www.ncbi.nlm.nih.gov/pmc/articles/PMC4148946/pdf/bmj.g4988.pdf

66. Das A, Friedman J, Kandpal E, Ramana GN V, Gupta RK Das, Pradhan MM, et al. Strengthening malaria service delivery through supportive supervision and community mobilization in an endemic Indian setting: an evaluation of nested delivery models. Malar J [Internet]. 2014;13(1):482. Available from: http://malariajournal.biomedcentral.com/articles/10.1186/1475-2875-13-482

67. Das VNR, Pandey RN, Pandey K, Singh V, Kumar V, Matlashewski G, et al. Impact of ASHA Training on Active Case Detection of Visceral Leishmaniasis in Bihar, India. PLoS Negl Trop Dis. 2014;8(5):1–5.

68. Shashikala N, Kulkarni P, Renuka M, Sunil D. Strategising the Educational Interventional Methods on Infant Feeding Practices : An Exploratory Study among Primary Care Workers in Rural Mysore. 2016;

69. Nyamathi A, Sinha S, Ganguly KK, Ramakrishna P, Suresh P, Carpenter CL. Impact of protein supplementation and care and support on body composition and CD4 count among HIV-infected women living in rural India: Results from a randomized pilot clinical trial. AIDS Behav. 2013;17(6):2011–21.

70. Nyamathi A, Ekstrand M, Salem BE, Sinha S, Ganguly KK, Leake B. Impact of Asha intervention on stigma among rural Indian women with AIDS. West J Nurs Res [Internet]. 2013;35(7):867–83. Available from: http://www.scopus.com/inward/record.url?eid=2-s2.0-84880472427&partnerID=tZOtx3y1

71. Nyamathi A, Salem B, Meyer V, Ganguly K, Sinha S, Ramakrishnan P. Impact of an ASHA Intervention on Depressive Symptoms among Rural Women Living with AIDS in India: Comparison of the Asha Life and Usual Care Program. AIDS Educ Prev. 2013;24(3):280–93.

72. Nyamathi A, Hanson AY, Salem BE, Sinha S, Ganguly KK, Leake B, et al. Impact of a Rural Village Women (Asha) Intervention on Adherence to Antiretroviral Therapy in Southern India. Nurs Res [Internet]. 2012;61(5):353–62. Available from: http://search.ebscohost.com/login.aspx?direct=true&db=aph&AN=82086152&site=ehost-live

73. Sebastian MP, Khan ME, Kumari K, Idnani R. Increasing postpartum contraception in rural India: Evaluation of a community-based behavior change communication intervention. Int Perspect Sex Reprod Health. 2012;38(2):68–77.

74. Dixit A, Khan ME, Bhatnagar I. Mainstreaming of Emergency Contraception Pill in India: Challenges and Opportunities. Indian J Community Med [Internet]. 2015;40(1):49–55. Available from: http://10.0.16.7/0970-0218.149271%5Cnhttp://search.ebscohost.com/login.aspx?direct=true&db=a9h&AN=100672766&site=ehost-live

75. Pala S, Kumar D, Jeyashree K, Singh A. Preliminary evaluation of the ASHA scheme in Naraingarh block, Haryana. Natl Med J India. 2011;24(5):315–6.

76. Saprii L, Richards E, Kokho P, Theobald S, Greenspan J, McMahon S, et al. Community health workers in rural India: analysing the opportunities and challenges Accredited Social Health Activists (ASHAs) face in realising their multiple roles. Hum Resour Health [Internet]. 2015;13(1):95. Available from: http://www.human-resources-health.com/content/13/1/95

77. Swain S, Swain P, Nair KS, Dhar N, Gupta S, Nandan D. A rapid appraisal of functioning of ASHA under NRHM in Orissa. Heal Popul Perspect Issues [Internet]. 2008;31(2):73–9. Available from: http://www.scopus.com/inward/record.url?eid=2-s2.0-60749127337&partnerID=40&md5=40e1a22cda022b403a8aac73e10da628

78. Engel N, Ganesh G, Patil M, Yellappa V, Pai NP, Vadnais C, et al. Barriers to point-of-care testing in India: Results from qualitative research across different settings, users and major diseases. PLoS One [Internet]. 2015;10(8). Available from: http://dx.doi.org/10.1371/journal.pone.0135112

79. Sidney K, Tolhurst R, Jehan K, Diwan V, De Costa A. “The money is important but all women anyway go to hospital for childbirth nowadays” - a qualitative exploration of why women participate in a conditional cash transfer program to promote institutional deliveries in Madhya Pradesh, India. BMC Pregnancy Childbirth [Internet]. 2016;16(1):47. Available from: http://www.pubmedcentral.nih.gov/articlerender.fcgi?artid=4779242&tool=pmcentrez&rendertype=abstract

80. Silan V, Kant S, Archana S, Misra P, Rizwan SA. Determinants of underutilisation of free delivery services in an area with high institutional delivery rate: A qualitative study. N Am J Med Sci. 2014;6(7):315–20.

81. Smith R, Menon J, Rajeev JG, Feinberg L, Kumar RK, Banerjee A. Potential for the use of mHealth in the management of cardiovascular disease in Kerala: a qualitative study. BMJ Open [Internet]. 2015;5(11):e009367. Available from: http://www.pubmedcentral.nih.gov/articlerender.fcgi?artid=4654349&tool=pmcentrez&rendertype=abstract

82. Jose R, Pisharady R, Benny P V., Nujum ZT, Rema Devi S, Varghese S, et al. Evaluation of non communicable disease control pilot programme of National Rural Health Mission in Thiruvananthapuram district. Clin Epidemiol Glob Heal [Internet]. 2015;3(1):17–23. Available from: http://dx.doi.org/10.1016/j.cegh.2013.08.004

83. Mishra A. The role of the Accredited Social Health Activists in effective health care delivery: evidence from a study in South Orissa. BMC Proc [Internet]. 2012;6(Suppl 1):1–2. Available from: http://search.ebscohost.com/login.aspx?direct=true&db=aph&AN=71341372&site=ehost-live

84. Nordfeldt C, Roalkvam S. Choosing Vaccination: Negotiating Child Protection and Good Citizenship in Modern India. Forum Dev Stud. 2010;37(3):327–47.

85. Awasthi S, Nichter M, Verma T, Srivastava NM, Agarwal M, Singh JV, et al. Revisiting community case management of childhood pneumonia: Perceptions of caregivers and grass root health providers in Uttar Pradesh and Bihar, Northern India. PLoS One. 2015;10(4):1–19.

86. Elazan SJ, Higgins-Steele AE, Fotso JC, Rosenthal MH, Rout D. Reproductive, Maternal, Newborn, and Child Health in the Community: Task-sharing Between Male and Female Health Workers in an Indian Rural Context. Indian J Community Med [Internet]. 2016;41(1):34–8. Available from: http://www.scopus.com/inward/record.url?eid=2-s2.0-84950342114&partnerID=tZOtx3y1

87. Fotso JC, Higgins-Steele A, Mohanty S. Male engagement as a strategy to improve utilization and community-based delivery of maternal, newborn and child health services: evidence from an intervention in Odisha, India. BMC Health Serv Res [Internet]. 2015;15 Suppl 1(Suppl 1):S5. Available from: http://www.pubmedcentral.nih.gov/articlerender.fcgi?artid=4464214&tool=pmcentrez&rendertype=abstract

88. Sharma R, Webster P, Bhattacharyya S. Factors affecting the performance of community health workers in India: A multi-stakeholder perspective. Glob Health Action. 2014;7(1):1–8.

89. Shrivastava A, Srivastava A. Measuring communication competence and effectiveness of ASHAs (accredited social health activist) in their leadership role at rural settings of Uttar Pradesh (India). Leadersh Heal Serv [Internet]. 2016;29(1):69–81. Available from: http://www.emeraldinsight.com/doi/10.1108/LHS-12-2014-0079

90. Kaphle S, Chaturvedi S, Chaudhuri I, Krishnan R, Lesh N. Adoption and Usage of mHealth Technology on Quality and Experience of Care Provided by Frontline Workers: Observations From Rural India. JMIR mHealth uHealth. 2015;3(2):e61.

91. Sundararaman T. Community health-workers: scaling up programmes. Lancet. 2007;369(9579):2058–9.

92. Praveen D, Patel A, Raghu A, Clifford GD, Maulik PK, Mohammad Abdul A, et al. SMARTHealth India: Development and Field Evaluation of a Mobile Clinical Decision Support System for Cardiovascular Diseases in Rural India. JMIR mHealth uHealth [Internet]. 2014;2(4):e54. Available from: http://www.ncbi.nlm.nih.gov/pubmed/25487047

93. Vashistha A, Kumar N, Mishra A, Anderson R. Mobile Video Dissemination for Community Health. Proc Eighth Int Conf Inf Commun Technol Dev - ICTD ’16 [Internet]. 2016;1–11. Available from: http://dl.acm.org/citation.cfm?doid=2909609.2909655

94. Srivastava A, Gope R, Nair N, Rath S, Rath S, Sinha R, et al. Are village health sanitation and nutrition committees fulfilling their roles for decentralised health planning and action? A mixed methods study from rural eastern India. BMC Public Health [Internet]. 2016;16(1):59. Available from: http://www.scopus.com/inward/record.url?eid=2-s2.0-84959364938&partnerID=tZOtx3y1

95. Balasubramaniam SS, Sarojini N, Khanna R. An investigation of maternal deaths following public protests in a tribal district of Madhya Pradesh, central India. Reprod Health Matters [Internet]. 2012;20(39):11–20. Available from: http://dx.doi.org/10.1016/S0968-8080(12)39599-2

96. Kumar S, Roy R, Dutta S. Scaling-up public sector childhood diarrhea management program: Lessons from Indian states of Gujarat, Uttar Pradesh and Bihar. J Glob Health [Internet]. 2015;5(2):020414. Available from: http://www.ncbi.nlm.nih.gov/pubmed/26682047%5Cnhttp://www.pubmedcentral.nih.gov/articlerender.fcgi?artid=PMC4676586

97. Gopalan SS, Mohanty S, Das A. Assessing community health workers’ performance motivation: a mixed-methods approach on India’s Accredited Social Health Activists (ASHA) programme. BMJ Open. 2012;2(5):e001557–e001557.

98. Pandey J, Singh M. Donning the mask: Effects of emotional labour strategies on burnout and job satisfaction in community healthcare. Health Policy Plan. 2016;31(5):551–62.

99. Bhatia K. Performance based incentives of the ASHA Scheme: Stakeholders’ Perspectives. Econ Polit Wkly. 2014;49(22):145.

100. Swaminathan P. The formal creation of informality, and therefore, gender injustice: Illustrations from India’s social sector. Indian J Labour Econ. 2015;58(1):23–42.

101. Nambiar D, Sheikh K, Verma N. Scale-up of community action for health: lessons from a realistic evaluation of the Mitanin program in Chhattisgarh, India. BMC Proc [Internet]. 2012;6; 5(Suppl 5):026. Available from: http://search.ebscohost.com/login.aspx?direct=true&db=aph&AN=82432027&site=ehost-live%5Cnhttp://search.ebscohost.com/login.aspx?direct=true&db=awn&AN=25520803&site=ehost-live

102. Nandi S, Schneider H. Addressing the social determinants of health: A case study from the Mitanin (community health worker) programme in India. Health Policy Plan. 2014;29:ii71-ii81.

103. Scott K, Shanker S. Tying their hands? Institutional obstacles to the success of the ASHA community health worker programme in rural north India. AIDS Care. 2010;22 Suppl 2(931159996):1606–12.

104. Mishra A. ‘Trust and teamwork matter’: Community health workers’ experiences in integrated service delivery in India. Glob Public Heal An Int J Res Policy Pract [Internet]. 2014;9(8):960–74. Available from: http://dx.doi.org/10.1080/17441692.2014.934877

105. Roalkvam S. Health governance in India: citizenship as situated practice. Glob Public Health [Internet]. 2014;9(8):910–26. Available from: http://www.pubmedcentral.nih.gov/articlerender.fcgi?artid=4166913&tool=pmcentrez&rendertype=abstract

106. Som M. Volunteerism to Incentivisation: Changing Priorities of Mitanins Work in Chhattisgarh. Indian J Gend Stud [Internet]. 2016;23(1):26–42. Available from: http://ijg.sagepub.com/cgi/content/abstract/23/1/26

107. Joshi S, George M. Healthcare through community participation: Role of ASHA. Econ Polit Wkly. 2012;47(10):70–6.

108. Eble A. Child mortality in rural India: How the ASHA program works, and how it might fail. In: Miklian J, Kolas A, editors. India’s Human Security: Lost debates, forgotten people, intractable challenges. Oxon: Routledge; 2014. p. 168–83.

109. Bhatia K. Community health worker programs in India: a rights-based review. Perspect Public Health. 2014;134(5):276–82.

110. Patel AR, Nowalk MP. Expanding immunization coverage in rural India: A review of evidence for the role of community health workers. Vaccine. 2010;28(3):604–13.

111. Liu A, Sullivan S, Khan M, Sachs S, Singh P, Lui A, et al. Community Health Workers in Global Health: Scale and Scalability. Mt Sinai J Med. 2011;78(3):419–35.

112. Singh D, Negin J, Otim M, Orach CG, Cumming R. The effect of payment and incentives on motivation and focus of community health workers: five case studies from low- and middle-income countries. Hum Resour Health [Internet]. 2015; Available from: http://dx.doi.org/10.1186/s12960-015-0051-1

113. Zulu JM, Kinsman J, Michelo C, Hurtig AK. Integrating national community-based health worker programmes into health systems: a systematic review identifying lessons learned from low- and middle-income countries. BMC Public Health [Internet]. 2014;14(987):1–17. Available from: http://onlinelibrary.wiley.com/o/cochrane/cldare/articles/DARE-12014059364/frame.html

114. Nandan D. National Rural Health Mission -- rhetoric or reality. Indian J Public Health. 2005;49(3):168–70.

115. Paul VK. Newborn healthcare in India: The road ahead. J Neonatol [Internet]. 2009;23(3):191–8. Available from: http://ovidsp.ovid.com/ovidweb.cgi?T=JS&CSC=Y&NEWS=N&PAGE=fulltext&D=emed10&AN=2011030949%5Cnhttp://lshtmsfx.hosted.exlibrisgroup.com/lshtm?sid=OVID:embase&id=pmid:&id=doi:&issn=0973-2179&isbn=&volume=23&issue=3&spage=191&pages=191-198&date=2009&title=Jou

116. Nyamathi A. Engaging Community Health Workers in HIV/AIDS Care: A Case Exemplar Among Rural Indian Women Living With AIDS. J HIV AIDS Soc Serv [Internet]. 2014;13(4):330–6. Available from: http://www.tandfonline.com/doi/abs/10.1080/15381501.2014.964536

117. Ray SK. National rural health mission -- opportunity for Indian Public Health Association. Indian J Public Health. 2005;49(3):171–4.

118. Kapil U. National rural health mission: Training of Health Functionaries. Indian J Pediatr. 2006;73:248.

119. Bajpai V, Saraya A. NRHM - The panacea for rural health in India: A critique. Indian J Public Heal Res Dev. 2013;4(1):241–4.

120. Mudur G. India launches national rural health mission. BMJ [Internet]. 2005;330(7497):920. Available from: http://www.pubmedcentral.nih.gov/articlerender.fcgi?artid=556363&tool=pmcentrez&rendertype=abstract

121. Shukla A. National rural health mission--hope or disappointment? Indian J Public Health [Internet]. 2005;49(3):127–32. Available from: http://www.ncbi.nlm.nih.gov/pubmed/16468275

122. Taneja DK. National Rural Health Mission - a critical review. Indian J Public Health. 2005;49(3):152–5.
