# Supplementary material for: Taking stock of 10 years of published research on the ASHA programme: examining India’s national community health worker programme from a health systems perspective
Source: Health Res Policy Syst. 2019 Mar 25;17:29. doi: 10.1186/s12961-019-0427-0 (PMC6434894; doi:10.1186/s12961-019-0427-0)
Supplement: Supplementary file 4 — Summary of research on the main ASHA programme. (DOCX 71 kb) [file 12961_2019_427_MOESM4_ESM.docx]

| **Additional file 4. Summary of research on the main ASHA program** | | | | |
| --- | --- | --- | --- | --- |
| Author (date) | Location | Research focus | Outcomes | Overall findings |
| Awasthi, Nichter, et al. (2015) | Multiple (Uttar Pradesh and Bihar) | To identify predisposing, enabling and service-related factors influencing treatment delay for community acquired pneumonia in rural communities | While the community did seek information from ASHAs on childhood illnesses, ASHAs had limited knowledge about the signs of community acquired pneumonia  and its management. | mixed |
| Bajpai & Saraya (2013) | National | To critically scrutinize the NRHM's failure to meet its stated objectives in the first six years | The health of the people is not a standalone phenomenon that can be improved through healthcare alone. It requires a comprehensive action plan encompassing food security, employment and poverty alleviation as well. The incentive structure of the ASHA program detracts from the activist vision for these CHWs. | negative |
| Balasubramaniam, Sarojini & Khanna (2012) | Madhya Pradesh | To investigate high number of maternal deaths in a tribal area of Madhya Pradesh | There was an absence of ANC despite high levels of anaemia, absence of skilled birth attendants, failure to carry out emergency obstetric care in obvious cases of need, and referrals that never resulted in treatment. The ASHA was underutilized, many families had not heard of her and ASHAs had large gaps in essential knowledge of basic obstetric complications. The authors question the policy of giving cash to pregnant women to incentivize institutional delivery without first ensuring quality of care and strengthening the facilities to cope with the increased patient loads. There is a lack of accountability, discrimination against and negligence of poor women, particularly tribal women, and a close link between poverty and maternal death. | negative |
| Bansal, et al. (2016) | Gujarat | To assess ASHA knowledge on core clinical skills for home based newborn care | Knowledge and skills of ASHA workers in this region were inadequate. Satisfactory skills were found in 52%, 61%, 43%, and 68% of ASHA workers for temperature measurement, hand washing, weight measurement, and kangaroo mother care, respectively. None of the participants demonstrated satisfactory skills in bag and mask ventilation. | negative |
| Bhandari, Mazumder, et al. (2012) | Haryana | To evaluate the Indian Integrated Management of Neonatal and Childhood Illness (IMNCI) programme | Implementation of the IMNCI resulted in substantial improvement in infant survival and in neonatal survival in those born at home. ASHAs ran women’s group meetings in every village every three months to raise awareness about newborn care practices. | positive |
| Bhargavi & A. Sharma (2014) | Delhi | To assess beneficiaries' awareness, coverage and satisfaction of ASHA maternal and child health services | The involvement of ASHAs was found to be useful and successful for an overall improvement in terms of increase in awareness, percentage of utilization, acceptance and satisfaction in maternal and child health services in Delhi. | positive |
| Bhatia (2014a) | National | To derive relevant policy implications to stem attrition and enable sustenance of large-scale CHW programs, accounting for the needs and rights of the CHWs | CHWs in three successive Indian national CHW programs have consistently asked for reforms in their service conditions, including increased remuneration. Despite an evolution in stakeholder perspectives regarding the rights of CHWs, service reforms are slow. Performance-based payments (which are being applied in the ASHA program) do not provide the financial security expected by CHWs. The two hallmark characteristics of CHWs, namely, their volunteer status and the flexibility of their tasks and timings, impede their rights. | negative |
| Bhatia (2014b) | Maharashtra | To understand how ASHAs and related stakeholders feel about their remuneration | Remuneration is a growing concern for ASHAs and their families. The government should provide fixed payments, beyond which task-based incentives should continue to be given, though at a revised rate. The current system of remuneration is making it difficult for ASHAs to meet their family's needs and community's expectations. Further, payment and reimbursement procedures need to be simplified. | negative |
| Dixit, Khan & I. Bhatnagar (2015) | National | To explore barriers to mainstreaming use of the emergency contraceptive pill (ECP) | Since 2011, ASHAs have been designated ECP providers, however no ASHAs were trained on ECPs, most do not receive regular supplies, and many are confused about the difference between oral contraceptive pills, ECPs and abortion pills. Over half the gynaecologists surveyed disapproved of allowing ASHAs to provide ECPs. | mixed |
| Eble (2013) | National | To summarize what we know about the efficacy of CHW programs and point out possible pitfalls that the government must avoid if it hopes to achieve the child mortality-related goals of the NRHM. | The logic for placing the burden of reducing rural mortality on ASHAs, who are far removed from medical knowledge, arises from (1) the idea that they are most invested in success as local women themselves, (2) the historical failure of top down models of care; and (3) that the majority of causes of mortality can be prevented through simple interventions. Challenges include: (1) convincing the ASHA herself about the practices she promotes; (2) that the ASHA promotes the correct messages; (3) that she can convince others, especially in relation to opposition from families, traditional practitioners, and the need for health system support. | mixed |
| Engel, et al. (2015) | Karnataka | To identify barriers to successful point-of-care testing, namely ensuring the completion of the test and treat cycle in the same encounter | In India, the onus is on the patient to ensure successful point-of-care testing (such as for malaria, TB, HIV or even pregnancy) amidst uncoordinated providers, including the ASHA, with divergent and often competing practices, in settings lacking material, money and human resources. Even if tests can be conducted on the spot and infrastructure challenges have been resolved, improved relationships among providers and between patients and providers are required for successful point-of-care testing. | negative |
| Fathima, et al. (2015) | Karnataka | To assess adherence to selection criteria in the recruitment of ASHA workers and to assess their performance against their job descriptions | ASHA workers were largely recruited as per pre-set selection criteria with regard to age, education, family status, income, and residence. The ASHA workers were found to be functional in some areas with scope for improvement in others. The role of an ASHA worker was perceived to be more of a link-worker/facilitator rather than a community health worker or a social activist. | mixed |
| Gopalan & Durairaj (2012) | Odisha (Orissa) | To evaluate the impact of Janani Suraksha Yojana on women's access to maternal healthcare, OOP spending and ASHA performance motivation | The number of institutional deliveries, ante-and post-natal care visits increased after the introduction of JSY (annual net growth of 18.1%, 3.6% and 5% respectively). The financial incentive covered only 25.5% of the maternal healthcare cost of the beneficiaries in rural areas and 14.3% in urban areas and induced fresh out-of-pocket spending for some mothers (including informal payments to providers). ASHAs were highly motivated by their incentive and were satisfied with mothers’ attitudes toward them. They were demotivated by poor transportation and late JSY payment and spent a lot of their incentive on food, transportation and medicine for mothers. ASHAs desired further training. | mixed |
| Gopalan, Mohanty & A. Das (2012) | Odisha (Orissa) | To assess ASHA performance motivation, determinants of their motivation, and ASHAs' perceptions and experiences on the factors affecting their motivation | ASHA motivation was highest for intrinsic job satisfaction (which includes happiness at having a job, satisfaction that the job is worthwhile, career and knowledge enhancement, feeling of accomplishment, awards), self-efficacy, interest and confidence in responsibilities and social responsibility and altruism. Motivation was lowest for community opinion on quality of public health care system, healthcare infrastructure, and workload. Moderate motivation was attributed to community recognition, training, support, supervision and incentives. There was no association established between their level of dissatisfaction on the incentives and the extent of motivation. | mixed |
| Hussain, Dandona & Schellenberg (2013 | Odisha (Orissa) | To assess health system readiness to deploy rapid diagnostic tests (RDTs) and ACT for malaria control across the State. | A high proportion of ANMs (80%) and ASHAs (77%) had the necessary level of knowledge to be able to use RDT for malaria diagnosis. The proportion of ASHAs trained on malaria case management was 89% (209/235). However, 71% of ANM and 55% of ASHAs usually referred falciparum-positive patients to the health facility for treatment. The major reason for referral being the non-availability of drugs at the ANM and ASHA level. | mixed |
| Joshi & M. George (2012) | Maharashtra | To evaluate the performance of ASHAs in the community and examine whether or not they can generate community participation. | ASHAs had very poor socio-economic status and primarily took up this work to bring in income for their families. Their work is skewed towards incentivised activities, particularly pregnancy related. There is a contradiction between the activist idea and performance based incentives. | negative |
| Kansal, Kumar & Kumar (2012) | Uttar Pradesh | To assess the influence of educational level on ASHA functioning | One third of ASHA had schooling up to class eight, 37% high school, 22% intermediate, and 10% graduate. Statistically significant association between educational level & practices undertaken by ASHA in the community. ASHAs educated only to class eight face difficulty in filling Village Health Index Register provided to them. The study recommends that if it is difficult to upgrade the minimum educational level for the appointment of ASHA, there is a strong need of regular training & monitoring of ASHA from other functionaries. Village Health Index Register should be simplified so that they can maintain the record properly. Otherwise, recruiting more educated women to become ASHAs is likely to improve the service delivery. | mixed |
| Kapil (2006) | National | To suggest improvements on the proposed ASHA training program | Training is expensive and time consuming. There is often too little evaluation done. ASHAs should be trained in the first two years so they have five years to perform. | NA |
| Kohli, Kishore, et al. (2015) | Delhi | To assess ASHA knowledge and practices for maternal health care delivery | Although ASHAs knowledge is good, insufficient monitoring and health system shortcomings limit their capacity to convert knowledge into practice. Most ASHAs were aware of their role in maternal health services: bringing women for ANC (95%), counselling for family planning (96%), and accompanying women for hospital delivery (89%). Most (87%) knew that iron tablets have to be taken for minimum 100 days during pregnancy and most (93%) reported that they maintained an ANC register. ASHAs reported challenges including a shortage of staff at health centers (16%), no transportation facilities (15%), no money for emergencies, and opposition from local dais (13% each). | mixed |
| Kori, Bhatia & Mishra (2015) | Madhya Pradesh | To assess ASHA knowledge and performance | Over half the ASHAs had very good or good knowledge of: their roles and responsibilities, breastfeeding, immunization, government nutrition (anganwadi) services, family planning and STDs/HIV/TB/malaria and drugs. Lower scores predominated on ANC, intranatal care, post natal care and newborn care. Only seven of 26 key danger signs in pregnancy, during delivery, and in the postnatal period, were widely known (over 80% of ASHAs reported them). There is a need to revise and update the knowledge of ASHA workers from time to time. | mixed |
| Kosec, et al. (2015) | Bihar | To identify the factors that predict effective delivery of immunization information and services, food supplements, pregnancy care information, and nutrition information.by ASHAs and AWWs, or receipt of services by households | About 35% of households reported receiving any of the four services. ASHA-related results: ASHAs receiving incentives for institutional delivery (OR = 1.52, CI = 0.99–2.33) was marginally associated with higher odds of receiving pregnancy care information, and ASHAs who maintained records of pregnant women was significantly associated with households receiving such information (OR=2.25, CI=1.07–4.74). Product-oriented incentives affect delivery of both product- and information-oriented services, although household factors are also important. | mixed |
| Kumar, Kaushik and Kansal (2012) | Uttar Pradesh | To identify the factors that influence the work performance of ASHAs in eastern UP | Few ASHA knew that motivating communities to construct toilets (16%) and proving medical treatment for minor ailments (23%) were part of their responsibilities. More knowledgeable ASHAs reported higher performance. Lower caste (SC) ASHAs reported poorer performance than higher caste (OBC, general) ASHAs, which was attributed to discrimination by higher caste community members. | mixed |
| Lui, et al. (2011) | National | To summarize evidence and recommendations on overcoming challenges in program design and sustainability when CHW programs are expanded at scale. | Community health worker programs have had a demonstrated impact on morbidity and mortality and show enormous promise for scale-up. Addressing a crisis in human resources for health at this scale is an achievement, but the healthcare system must be prepared to receive, manage, and sustain an influx of workers when considering scale-up. | mixed |
| Malaviya, et al. (2013) | Bihar | To assess ASHA and ANM knowledge on visceral leishmaniasis (VL), experiences with VL, and preparedness for further involvement | ANMs and ASHAs know the presenting symptoms of VL and how it is diagnosed but are not aware of the recommended treatment. Few are involved in VL control. They are well organised, have strong links to the primary healthcare system and are ready to get more involved in VL control. | mixed |
| Malini, et al. (2008) | Odisha (Orissa) | To assess and evaluate JSY, reasons for non-utilization, perception and awareness of utilizers and non-utilizers, and involvement of ASHAs and other frontline providers | There was a lack of orientation given to staff other than ASHAs on JSY. ASHAs played a major role in motivating institutional deliveries in two-thirds of utilizers. Most utilizers expressed problems of communication and transportation. Non-availability of 24/7 facilities and lack of staff were major deterrents. | Mixed |
| Mishra (2012) | Odisha (Orissa) | To assess the role of ASHAs in promoting immunisation, referring and escorting for reproductive and child healthcare and facilitating access to quality health care | There was very limited community involvement in selecting ASHAs and ASHAs oriented themselves with the health system rather than the community. The ASHA-community relationship was stronger when ASHAs offered curative services for minor ailments. ASHAs strongly promoted prenatal check ups and institutional delivery, largely driven by their interest in receiving the JSY incentive. Immunization coverage increased due to regular immunization days. ASHA coordination meetings focused more on the submission of health records than discussing field experiences. | mixed |
| Mishra (2014) | Odisha (Orissa) | To provide empirical evidence on how integration of health care operated at the grassroots, through exploring community health workers' everyday experiences | For frontline health workers (including ASHAs), the notion of integration goes well beyond a technical lens of mixing different health services. They perceive ‘teamwork’ and ‘building trust with the community’ (beyond trust in health services) to be critical components of their practice. However, the comprehensive NRHM primary health care ideology is in constant tension with the exigencies of narrow indicators of health system performance. Monitoring mechanisms, the privileging of statistical evidence over field-based knowledge, and the highly hierarchical health bureaucratic structure that rests on top-down communications mitigate efforts towards sustainable health system integration. | mixed |
| Mishra, Kar & Satapathy (2015) | Odisha (Orissa) | [ASHA specific] To identify community members’ sources of information on mass drug administration for filariasis | ASHA specific findings: Out of the total 300 respondents of surveyed households, the major source of information regarding mass drug administration for filariasis was ASHA (47%), followed by AWW (29%) households. Television was the source of information in 5%) households and ANM in 2% households. | positive |
| Mohan, et al. (2011) | National | To assess the progress of integrated management of neonatal and childhood illness (IMNCI) in India, identify programme bottlenecks, and assess the effect on coverage of key newborn and childcare practices | More than 200,000 CHWs and first line health workers were trained 2005-9 across 223 districts. Of the reported births (n=1,102,573), 66% were visited by a trained worker within 24 hours, and 6% were visited three times within 10 days. Poor supervision and inadequate essential supplies affected the performance of trained workers. During 2004-2008, 12 early-implementing districts had covered most key newborn and child practice indicators compared to the control districts; however, the difference was significant only for care-seeking for acute respiratory infection (net difference: 17.8%; 95% CI 2.3 - 33.2 p<0.026). Based on the early experience of IMNCI implementation in different states of India, measures need to be taken to improve supportive supervision, availability of essential supplies and monitoring of the programme if the strategy has to translate into improved child survival in India. | mixed |
| Mony & Raju (2012) | Karnataka | To assess the operation of the ASHA program, including the main tasks being carried out by ASHAs and their reach to marginalized households. | ASHAs mostly work as link workers and community health workers, and to only a small extent as social activists. Within the domain of their link worker role, through their home visits to the households of community members they have contributed to improvements in the basic antenatal care and also in increasing the number of institutional deliveries and immunisation. Marginalized households are inadequately covered. | mixed |
| Mudur (2005) | National | To critically reflect on newly launched NRHM | Health activists say that far more funding is needed. The mission appears to rely on funds earlier spent on reproductive and child health. ASHAs should have been allotted reasonable compensation but are instead being implemented as volunteers. | NA |
| Nambiar, Sheikh, & Verma (2012) | Chhattisgarh | To understand the role of the Chhattisgarh State Health Resource Centre (SHRC) as a technical agency whose genesis and mandate is closely interlinked with the Mitanin program | Chhattisgarh’s new statehood in 2000 presented a context of strategic public sector system-building where health and community action were given priority, albeit in a larger context of tribal alienation and lack of women in statecraft. The creation of the SHRC institutionalized plural governance and collaboration between public and private stakeholders in supporting Mitanins, combining ‘technical’ competencies with ‘socio-political’ empowerment and mobilization. The Mitanin program has been successfully sustained through policymaker turnover and across multiple elections. Mitanins grassroots action has led the SHRC to develop career pathways and additional spaces for local health action. The SHRC has high retention of functionaries, which enhances linkages to local rights-based (tribal, food) campaigns. | positive |
| Nandan (2005) | National | To consider the NRHM's potential, including to improve community-level relationships and health care access | The NRHM holds great promise and the ASHA will play an important role, including in improving communication between socio-cultural clusters at the village level. Nonetheless, within-community divides are strong and the ASHA may not be able to address them. Community mobilizers, as have been used in other programs should be considered, as they can help strengthen the program through a cluster community approach that can coordinate, bring about health related behavior change, decrease "social delays" causing maternal and child mortality, and increase feelings of participation and ownership. | NA |
| Nandi & Schneider (2014) | Chhattisgarh | To document how and why the Mitanins have been able to act on the social determinants of health, describe the catalysts and processes involved and the enabling programmatic and organizational factors | Action on social determinants involved raising awareness on rights, mobilizing women’s collectives, revitalizing local political structures and social action targeting both the community and government service providers. Through these processes, the Mitanins developed identities as agents of change and advocates for the community, both with respect to local cultural and gender norms and in ensuring accountability of service providers. The factors underpinning successful action on social determinants include the original vision of the programme, how this intent was carried through into all aspects of programme design, Mitanin identification with village women, on-going training and support, and the relative autonomy of the programme. | positive |
| Nordfeldt & Roalkvam (2010) | Multiple (Uttarakhand & Rajasthan) | To understand why immunization rates in India remain low | There are three interfaces in rural vaccination programmes: between different knowledge systems; between social classes; and between government and citizens. The ASHA is deployed at all three of these interfaces. Despite a new language of community participation under the NRHM, reaching top-down targets remains the ASHAs’ main objective. Furthermore, information strategies present vaccination as the moral choice according to national needs, catering to discourses in Indian society ultimately legitimizing social inequalities. Choosing to vaccinate becomes a statement of belonging to the modern, implying a high moral status. Marginalized groups, conversely, are blamed for not being part of that moral community. Non-participation in the programme may represent a wish for dignity and a moral community of one’s own. A focus on preventive health care to reach all parts of the population is required: quality of delivery, local knowledge and structural constraints must be addressed. | negative |
| Padda, et al. (2013) | Punjab | To evaluate the role of ASHA workers in rural maternal health by making an urban-rural comparison | Maternal and child health services delivery improved after the inception of the ASHA program in rural communities. A higher percentage of rural versus urban women registered their pregnancy in the first trimester (86 v. 55%), received at least three ANC visits (91 v. 82%), received tetanus toxoid injection (86 v. 68%) and IFA (64 v. 46%) and, among those with home births, were visited by a health worker within 24 hours of delivery (84 v. 50%).. However, a lower percentage of rural versus urban women had institutional deliveries (86 v. 93%) and, among those with institutional births, received home visits within 48 hours (68 v. 37%). | positive |
| Pala, Kumar, et al. (2011) | Haryana | To evaluate the functioning of ASHAs in Naraingarh block and ascertain the opinion of the community about this new cadre of health workers | The ASHA scheme in the initial phases has many operational problems and the sustainability of these workers is also unclear. Of the 34 ASHA posts, 3 were not yet filled and 3 ASHAs had quit because (i) the monetary incentive was too little, (ii) there were difficulties in doing house-to-house survey because of caste issues, and (iii) the husband objected to house-to-house visits. Only 13 had received induction training and none had received on-the-job or refresher training. Most sarpanches were unaware of the program and no village health committees were functioning. Only 2 ASHAs felt that the amount they received was satisfactory. Medical kits were supplied irregularly and ASHAs lacked money to transport pregnant women to health facilities. | negative |
| Pandey & M. Singh (2016) | Uttarakhand | To investigate the link between surface and deep-level emotional labour, burnout and job satisfaction in ASHAs | Surface and deep-level emotional labour are two different strategies for performance of emotional labour in community health care. Surface-level emotional labour is associated with higher job satisfaction, and burnout partially mediates this relation. Deep-level emotional labour is associated with lower job satisfaction, and burnout fully mediates this relation. Qualitative post hoc analysis based on interviews with 10 ASHAs found that surface-level emotional labour was a more desirable strategy for effective and efficient performance. | mixed |
| Panigrahi, Mohapatra & K. Mishra (2015) | Odisha (Orissa) | To assess awareness of and perceptions about the village health and nutrition day (VHND) | While most ANMs and anganwadi workers considered health awareness a key component of the VHND, 52% of ANMs and 41% of AWWs had misconceptions about additional roles and responsibilities. ASHAs called beneficiaries together for VHNDs but few beneficiaries knew the purpose of the VHND (24%) and reasons they were referred for further care (8%). | negative |
| Patel & Nowalk (2010) | National | To examine the potential contribution of CHWs toward strengthening immunization services in rural India | While the limited number and quality of available studies make it difficult to directly compare CHW interventions to other strategies for improving immunization coverage, it is clear that CHWs make diverse contributions toward strengthening immunization programs. Incorporation of evidence-based strategies for CHW selection, retention, and training is critical for the success of India’s immunization program. In addition, there is growing need to develop efficient mechanisms for monitoring children’s vaccination status to generate actionable feedback and identify cost-effective strategies. | NA |
| Paul (2009) | National | To identify strategies to improve neonatal healthcare in India | Most of the evidence-based interventions are reflected in the programs, but the coverage levels are low due to poor implementation. Action is required at the home and community level, as well as at the outreach and facility levels in rural and urban settings. ASHAs must be engaged in home care of neonates. Effective programming would require higher budgets, decentralized planning, managerial support, proper monitoring and a massive human resources capacity development. Community mobilization is essential, for which panchayati raj institutions can play an important role, as is ongoing advocacy. | NA |
| Persai, Panda & Mathur (2015) | Multiple (Gujarat and Andhra Pradesh) | To explore whether CHWs such as ASHAs can be utilized as a resource for informing and educating community on tobacco and its harmful effects; to capture perceptions and practices of ASHAs regarding tobacco control | ASHAs linked tobacco usage to diseases such as respiratory problems, lung cancer, tuberculosis, and oral disease. However, ASHA provision of information on tobacco‐related diseases is suboptimal: only one‐third of ASHAs reported informing all patients about the harmful health effects of tobacco, whereas more than half reported providing information only to patients suffering from specific illnesses. ASHAs who reported having received training in tobacco control were about two times more likely to give information on effects of tobacco on respiratory diseases (OR: 1.5; CI: 1.1–2.4) and adverse reproductive outcomes (OR: 2.1; CI: 1.1–20.2). | mixed |
| Prasad (2009) | Assam | To assess whether the activities of the malaria control program were taking place in an effective and judicious manner | The malaria control programme has been seriously jeopardized by improper implementation of vector control measures, lack of adequate professional support and varied commitment on the part of the state government. None of the 22 ASHAs surveyed were involved in anti-malaria activities. Indiscriminate use of rapid diagnostic test kits yielded poor and unsatisfactory response. | negative |
| Ramadurg, et al. (2015) | Karnataka | To assess CHW knowledge and management of pre-eclampsia | Knowledge on the causes of pre-eclampsia was limited among ANMs, ASHAs and staff nurses. Psychological explanations of hypertension were most commonly reported: stress, tension, and fear. The most common explanation for eclampsia was not receiving a tetanus vaccination. Despite some common misperceptions regarding aetiology, these CHWs demonstrated a good grasp of the potential consequences of hypertension in pregnancy. | mixed |
| Ray (2005) | National | To present the Indian Public Health Association's (IPHA) suggestions on how best to implement the NRHM | The IPHA supports the vision of the ASHA program, and suggests that stakeholder (panchayati raj, ICDS, etc.) perspectives should guide ASHA selection and roles. | NA |
| Roalkvam (2014) | Rajasthan | To explore how women, as mothers and health workers, organise themselves in relation to rights and identities | The rights of citizenship are not solely contingent upon the existence of legally guaranteed rights but also significantly on the social conditions that make their effective exercise possible. This implies that while citizenship is in one sense a membership status that entails a package of rights, duties, and obligations as well as equality, justice, and autonomy, its development and nature can only be understood through a careful consideration and analysis of contextually specific social conditions. | mixed |
| Sagare, et al. (2012) | Maharashtra | To understand ASHA knowledge, attitude and practices on TB | ASHAs had good knowledge (Mean score = 6.58 out of 10) on tuberculosis and DOTS. All knew that tuberculosis can be cured with prompt treatment, 67% ASHAs knew that TB diagnosis and DOTS are free of cost, 95% said they would to a health facility if they thought that they themselves had symptoms of tuberculosis. 88% had a favourable attitude towards tuberculosis patients. However, gaps in the knowledge of BCG vaccination and major symptoms of pulmonary tuberculosis were observed. | mixed |
| Sahu, Rao & Dash (2016) | Odisha (Orissa) | To assess the performance of ASHAs posted during 2006 to 2009 in diagnosis of malaria and treatment | The passive system of early malaria diagnosis and prompt treatment operated by ASHAs in tribal areas is working successfully and needs to be further strengthened. The majority of the households (60%) visited ASHAs for fever treatment and among them 49% were satisfied with the diagnosis and anti-malarials treatment. 70% of ASHAs interviewed had Rapid Diagnostic Kits and 32% had anti-malarial drugs at the time of interview. | mixed |
| Salve, Babu, et al. (2014) | Haryana | To assess attitudes towards mental illness among health workers (including ASHAs) and community leaders | Community members reported socially restrictive, pessimistic and stereotyping attitudes towards mentally ill people. ASHAs and health care providers reported similarly stereotyping attitudes. None of the stakeholders reported stigmatizing attitude. ASHAs and other providers need training on the spectrum of mental illness. Community leaders would benefit from increased awareness of the bio-medical nature of mental illness. | mixed |
| Saprii, Richards, et al. (2015) | Manipur | To understand how ASHAs realize their multiple roles | ASHAs are valued for their contribution towards maternal health education and for their ability to provide basic biomedical care, but their role as social activists is much less visible as envisioned in the ASHA operational guideline. Access by ASHAs to fair monetary incentives commensurate with effort coupled with improved health system functionality will strengthen the role of ASHAs. | mixed |
| Saxena, Kakkar & Semwal (2012) | Uttarakhand | To find out the biosocial profile of ASHA and services provided by them | The majority of ASHAs consider care of pregnant women, vaccination and family planning as their prime services. 42% reported that they think this work can pave their ways for future employment. Few knew of their role as motivator and activist. | mixed |
| Saxena & Kumari (2014) | Uttarakhand | To identify ASHA knowledge and practices with respect to Infant and Young Child Feeding (IYCF) issues, particularly breastfeeding | Although ASHAs have high knowledge of IYCF, actual practice is poorer. ASHAs require skill building especially in the area of breastfeeding options for working women as well as complementary feeding. While 98% of ASHAs had complete and correct information about exclusive breastfeeding, only 38% ASHAs were aware that breastfeeding should be started within four hours in children delivered by caesarean section. 18% reported to be able to motivate mothers to practice exclusive breastfeeding, with failure linked to insufficient milk (55%), caesarean (20%), and pressure from elders in the family to supplement. Only 45% of ASHAs knew the correct timing of initiation of complementary feeding; 58% ASHAs had introduced complementary feeding at the seventh month in their children. Bottle feeding had been practiced by about 33% of ASHAs in the past. | mixed |
| Saxena et al. (2015) | Uttarakhand | To analyse the preparedness of ANMs, ASHAs, AWWs and PRI members to provide services at Village Health and Nutrition Days | Micro-plans or beneficiary checklists had been prepared by 86% of ANMs, 85% ASHAs and 33% of AWWs. Anganwadi centres were often unclear (42%), without clean drinking water (71%), without privacy for ANC (88%) and without required instruments (50%). Only 77% ANMs, 55% of ASHAs and 44% of AWW had participated in any VHND meetings in last three months. None of the VHND sites had displayed timings and information regarding services. | mixed |
| Scott & Shanker (2010) | Uttarakhand | To investigate contextual features of the programme that are hindering the ASHAs’ capacity to increase quantitative health outcomes and act as cultural mediators and agents of social change | ASHAs were institutionally limited by: (1) the outcome-based remuneration structure; (2) poor institutional support; (3) the rigid hierarchical structure of the health system; and (4) a dearth of participation at the community level. Progressive policy on CHW programmes must be backed up by concrete institutional support structures to enable CHWs to fulfil their role. | mixed |
| Sharma, Webster & Bhattacharyya (2014) | Rajasthan | To understand the coordination between the ASHAs and their co-workers (ANMs and AWWs) | ASHA motivation and performance are affected by personal (e.g. education), professional (e.g. training, job security), and organisational (e.g. infrastructure) factors along with others that emerge from external work environment. In order to improve the performance of ASHAs, apart from taking corrective actions at the professional and organisational front on a priority basis, it is equally essential to promote cordial work relationships amongst ASHAs, ANMs and AWWs. | mixed |
| Shrivastava, A. & Srivastava (2016) | Uttar Pradesh | To find out ASHA communication competence and effectiveness while working as leaders with groups in the rural setting | ASHAs need to be sensitized on the critical role of effective communication. Greater investment is required in ASHA capacity building for health communication. The trainings being imparted to ASHAs have to be strengthened in terms of communication skills. | mixed |
| Shrivastava, S.R. & Shrivastava (2012) | Maharashtra | To evaluate knowledge, attitudes and practices of ASHA workers in relation to child health | Gaps persist in ASHA knowledge of various aspects of child health. The majority (67%) of ASHAs were not aware of the correct preventive measures for vitamin A deficiency. 20% did not feel the need to refer a child with diarrhoea who is unable to drink or breast feed, 24% did not know to refer a child with fast breathing and 50% did not know to refer a baby crying for more than 3 hours following immunization. Monthly meetings and periodical refresher training should be used to reinforce child health knowledge, especially on high risk cases requiring prompt referral. | mixed |
| Shukla, Abhay (2005) | National | To critically reflect on newly launched NRHM | Jan Swasthya Abhiyan (the Indian arm of the People’s Health Movement) calls for the newly launched NRHM to be oriented around strengthening an integrated public health system and empowering communities to be involved in the planning and utilization of these systems in a rights-based framework. | mixed |
| Shukla, Amit and Bhatnagar (2012) | Uttarakhand | To assess the knowledge, attitude, practices, hindrances and motivation factors among ASHAs regarding pregnancy-related conditions | Utilisation of ASHA for ANC was high but lower for delivery-related and postnatal care services. ASHAs have optimal knowledge of expected work and are the major source of information and support for pregnancy-related services. | mixed |
| Sidney, Tolhurst, et al. (2016) | Madhya Pradesh | To explore why women participate (or not) in the JSY program. | Women’s increased participation in the program reflects a shift in social norms, driven by social pressure from ASHAs to deliver in a health facility, and a growing individual perception of the importance for ‘safe’ and ‘easy’ delivery. While the incentive was an important influence on many women’s choices, others did not perceive it as an important consideration in their decision to deliver in a health facility. Many women reported procedural difficulties in receiving the benefit and high out-of-pocket expenditures at the facility. Non-participation was often unintentional and caused by personal circumstances, poor geographic access or driven by a perception of poor quality of care provided in program facilities. | mixed |
| Sidney, Diwan, et al. (2012) | Madhya Pradesh | To assess recently delivered mothers' knowledge of and participation in the JSY program (including receipt of the cash incentive), place of delivery, delivery type, ANC visits, role of ASHA in facilitating the delivery, and infant and maternal outcomes | The majority of deliveries (76%) took place within the JSY program; 81% of all mothers below poverty line delivered in the program. 90% of the women had prior knowledge of the program. Most program mothers reported receiving the cash incentive within two weeks of delivery. The ASHA’s influence on the mother’s decision on where to deliver appeared limited. Women who were uneducated, multiparious or lacked prior knowledge of the JSY program were significantly more likely to deliver at home. | mixed |
| Silan, et al. (2014) | Haryana | To understand, from the ASHAs’ viewpoint, reasons for underutilization of zero expense delivery services provided in government health facilities | Underutilization of government health facilities for delivery services was attributed to a lack of quality care, disrespectful behaviour by hospital staff, poor transportation facilities, and frequent referrals to higher centres. | NA |
| Singh, et al. (2015) | National | To examine the effect of payment and incentives on motivation and focus of community health workers in five countries | Both volunteer and remunerated CHWs are potentially effective and can bring something to the health arena that the other may not. Well-trained, supervised volunteers and full-time CHWs who receive regular payment, or a combination of both, are more likely to engage the community in grass-roots health-related empowerment. Programmes that utilize minimal economic incentives to part-time CHWs tend to limit their focus, with financially incentivized activities becoming central. They can, however, improve outcomes in well-circumscribed areas. In order to maintain benefits from different approaches, there is a need to distinguish between CHWs that are trained and remunerated to be a part of an existing health system and those who, with little training, take on roles and are motivated by a range of contextual factors. | mixed |
| Sinha, Kaur, et al. (2014)^71^ | Haryana | To assess Home Based Post Natal Newborn Care knowledge, attitudes and practices among mothers and ASHAs | There is a gap between knowledge and practice among mothers counselled by ASHAs. Overall, 60% of mothers adopted less than three safe practices. Wrapping newborns and delayed bathing were better adopted than cord care, safe breastfeeding, hand washing, kangaroo care and eye care. Practices were influenced by cultural beliefs, traditional birth attendants, lack of supervision by ANM, and delayed referral and transportation. | mixed |
| Smith, et al. (2015) | Kerala | To assess the potential for using mHealth in cardiovascular disease (CVD) management in Kerala by exploring: experiences and challenges of current CVD management; current mobile phone use; and expectations of and barriers to mobile phone use in CVD management. | Patients had low knowledge of CVD and high CVD-risk lifestyles with low implementation of primary prevention measures. ASHAs mainly used their mobile phones for phone calls. mHealth was considered capable of improving ASHA access to healthcare knowledge; providing reminders of appointments, medication and lifestyle changes; saving time, money and travel; and improving ASHA job efficacy. | NA/ Formative |
| Som (2016) | Chhattisgarh | To understand Mitanin work and programme implementation before and after incentives were announced. | While the Mitanin programme had hoped that the community would pay Mitanins, in the initial couple of years this did not happen and Mitanins were not compensated. Later, activity-oriented payment was started, leading to incentivisation of their activities. Mitanins were supposed to raise the community’s concerns and demands as a community representative and organiser. However this role was not met and she became a very low paid, honorary worker of the government health service system. | negative |
| Srivastava, Aradhana et al. (2016) | Multiple (Jharkhand and Odisha) | To assess village health, sanitation and nutrition committee (VHSNC) composition, training of members, fund availability and activities, and understand members’ perception of their roles and functions, and levels of participation in planning and activities | VHSNCs comprised equitable representation from vulnerable groups and more than 75% of the members were women. Less than 1% of the members had received any training and supervision of committees by district or block officials was rare. VHSNCs focused on strengthening village sanitation, conducting health awareness activities, and supporting medical treatment for ill or malnourished children and pregnant mothers. 62% of the committees monitored community health workers, 7% checked sub-centres and 2% monitored drug availability with ASHAs. ASHAs and AWWs were conveners and record keepers. Key challenges included irregular meetings, members’ limited understanding of their roles and responsibilities, restrictions on planning and fund utilisation, and weak linkages with the broader health system. | negative |
| Srivastava, D.K. et al. (2009) | Uttar Pradesh | To study the functioning of ASHA in the community with special focus on the interface with the community and service providers | ASHAs were primarily motivated by incentives (82%) and getting a government job (67%). Most (87%) of ASHAs got support from their supervisor in solving problems and majority (95%) were satisfied with their supervisors. All the ASHAs were accepted well in the community and were acting as a good link between the community and health providers. Although accepted by the community, ASHAs need regular training, support and cooperation from other functionaries. | mixed |
| Sundararaman (2007) | Chhattisgarh | To reflect on scale up of programs | Core successes of the Mitanin program include sustained political relevance and increased women’s participation. The program is likely linked to decreased rural infant mortality. Program features central to success include: state government partnership with civil society; the development of a female middle-level supervisor and trainer cadre; and evolution of Mitanin roles to include child survival nutrition counselling and essential newborn care as well as rights-based activities that enabled access to basic public services as fundamental entitlements to be secured through women’s empowerment and community action. | mixed |
| Sundararaman, Ved, et al. (2012) | Multiple (Andhra Pradesh, Assam, Bihar, Jharkhand, Kerala, Orissa, Rajasthan, West Bengal) | To understand the evolution of the program, perspectives, and experiences of key stakeholders in specific context; to assess the functionality of ASHA in relation to her effectiveness in bringing health outcomes; and to review the quality of key mechanisms that constitute the program. | States have adapted aspects of the ASHA guidelines. 75% of pregnant women across the states received services from ASHAs. For an ASHA to be effective, all three roles (link worker, care provider and activist) are important and complementary in nature. The functionality of ASHAs in one role is clearly linked with better outcomes in other two roles. Prioritization of only the link worker function fails to make full use of her potential for child survival and reduces her ability to reach marginalized communities. Various state managers held diverse program theories that emphasized different operating mechanisms and in turn influenced program outcomes. Beyond provision of cash incentives, greater support should be given to competency based training, health rights, an adequate supply of medicines, and mentoring and motivation. | positive |
| Swain, et al. (2008) | Odisha (Orissa) | To understand the functioning of the ASHAs in the community and then suggest strategies for improvement. | Women in self help groups were involved in ASHA selection, making the process more transparent and the selected ASHAs more acceptable to the community. Community members accept ASHAs as a link between them and healthcare sector but also expect significant non-health sector work from ASHAs, such as help in getting old age pensions. There is good supportive monitoring system to supervise and facilitate the work of the ASHAs. The majority of the ASHAs are catering to a population of more than 1,000 in Orissa, and due to hilly geographical terrain, ASHAs fail to visit some hamlets. ASHAs’ distribution of medicine catalyses community acceptance but about a quarter of the ASHAs have not received medicine kits, and those with kits do not receive complete stocks. Non-availability of transport to visit pregnant women is a major problem. Lack of communication or unwillingness on the part of the beneficiaries to inform the ASHAs lead to loosing her incentive. Majority of the ASHAs are not getting incentives on time. | positive |
| Swaminathan (2015) | Multiple (Rajasthan & Maharashtra) | To critically explore the affect of India's approach to "doing development" on women's work | The state has instituted several welfare programmes, including the ASHA program, that, in the guise of ‘empowering’ women, has feminised responsibility and employed local women without designating them as workers. Women therefore must work for development, rather than development enabling women to procure ‘decent’ employment. The traditional hypothesis that the informal sector is a transient phenomenon and would disappear by getting absorbed in the formal sector over the years does not hold true. The informal nature of employment in the social sector has been formalised in several ways, such that while services are being delivered under the formal health/education sector, some of the personnel (mainly women) employed to deliver these services are informally employed within the same sector. | negative |
| Taneja (2005) | National | To critically reflect on newly launched NRHM | The ASHA program is an improvement over the earlier Community Health Guide Scheme. The ASHA program is similar in concept to the Community Health Guide scheme launched in 1977, however, it takes care of many of drawbacks of earlier scheme: ASHAs must be women, married, resident of the village, and educated up to 8th standard (against earlier 6th standard). Selection is also more embedded in the community. | NA |
| Thacker, et al. (2013) | Multiple (Uttar Pradesh & Bihar) | To assess attitudes, barriers and practices associated with polio eradication efforts. | More than 95% of ANMs and ASHAs agreed that polio supplementary immunization campaigns helped in increasing acceptance of all vaccines. The majority of ANMs (60–70%) and ASHAs (56–71%) believed that polio immunization activities benefitted or greatly benefitted other activities they were carrying out. Less than 5% of ANMs and ASHAs felt they were very likely to face resistance when promoting or administering polio vaccine. | positive |
| Verma & Rao (2014) | Multiple (Bihar, Uttar Pradesh & West Bengal) | To identify relevant factors that are preventing active participation of women and suggest corrective steps | Only 5% of ASHAs had participated in leprosy related work. Stringent steps are needed to re-orient and encourage ASHAs to undertake leprosy related work. Rural Indian women (non-ASHAs) are keen to play an important role in the national leprosy eradication program, with minimal support from the government and non-governmental agencies in a truly community-based approach. | negative |
| Vikram, Sharma & Kannan (2013) | Delhi | To identify the beneficiary level factors of utilization of JSY scheme in urban slums and resettlement colonies of the trans-Yamuna area of Delhi | While most (71%) women interviewed had an institutional delivery, only 27% benefited from JSY and only 15% received cash benefits from JSY. Belonging to Hindu religion and having had more than 6 antenatal check ups were the significant predictors of availing the benefits of JSY. There is a need to improve the awareness among urban slum population about the utilization of JSY scheme. | negative |
| Wagner, Bettampadi, et al. (2016) | National | To measure the impact of increased use of ASHAs on rural immunization coverage | Across the 267 districts in 21 states studied, 41% of villages had ASHA workers in DLHS-3, compared to 78% in DLHS-4. From DLHS-3 to DLHS-4, the average district-level coverage changed from 93% to 89% for BCG, 78% to 80% for polio vaccine, 81% to 79% for measles vaccine, and 63% to 51% for full vaccination, and remained at 78% for DPT. Greater than median increases in ASHA presence (≥30%) within a district were associated with 1.733 greater odds of increase in DPT coverage (95% CI: 1.036, 2.900) and 2.042 greater odds of increase in measles vaccine coverage (95% CI: 1.215, 3.430). Expanded ASHA coverage was not significantly associated with changes in BCG, polio vaccine, or full vaccination coverage. | mixed |
| Zulu, Kinsman, et al. (2014) | National | To examine factors that may influence integration of national CBHW programmes | Four programmes, from Brazil, Ethiopia, India and Pakistan, were examined. Factors that facilitated the CHW program integration process included the magnitude of countries’ human resources for health problems and the associated discourses about how to address these problems; the perceived relative advantage of national CHWs with regard to delivering health services over training and retaining highly skilled health workers; and the participation of politicians and community members in programme processes, with the result that they viewed the programmes as legitimate, credible and relevant. Integration of programmes within the existing health systems enhanced programme compatibility with the health systems’ governance, financing and training functions. Factors that inhibited the integration process included a rapid scale-up process; resistance from other health workers; discrimination of CHWs based on social, gender and economic status; ineffective incentive structures; inadequate infrastructure and supplies; and hierarchical and parallel communication structures. | mixed |
