# Supplementary material for: Taking stock of 10 years of published research on the ASHA programme: examining India’s national community health worker programme from a health systems perspective
Source: Health Res Policy Syst. 2019 Mar 25;17:29. doi: 10.1186/s12961-019-0427-0 (PMC6434894; doi:10.1186/s12961-019-0427-0)
Supplement: Supplementary file 5 — Summary of smaller scale interventions that engaged ASHAs. (DOCX 44 kb) [file 12961_2019_427_MOESM5_ESM.docx]

| Additional file 5: Summary of smaller scale interventions that engaged ASHAs | | | | | | |  |
| --- | --- | --- | --- | --- | --- | --- | --- |
| Author (date) | Location | Intervention | | | | Outcome | Overall findings |
| **Child health (n=15)** | | | | | | |  |
| Kumar, Roy & Dutta (2015) | Gujarat, Uttar Pradesh, Bihar | **Diarrhea Alleviation through Zinc and ORS Therapy (DAZT) & Reducing Deaths from Diarrhea**: a 3 year intervention to improve management of childhood diarrhea: (1) health worker (including ASHA) training; (2) service delivery through CHWs expanded; (3) supportive supervision to CHWs; (4) ensuring supplies; and (5) conducting monitoring and evaluation to improve diarrhea treatment with oral rehydration salts (ORS) and zinc. | | | CHWs had sustained knowledge and have treated more than three million children under five years with diarrhea, of which 84% were treated with both zinc and ORS. Authors conclude that it is feasible and viable to introduce and scale–up zinc and ORS for childhood diarrhea treatment through the public sector. Community–based service delivery, timely and adequate supplies, trained staff and pro–active engagement with government is essential for program success. | | positive |
| Lamberti, Christa, et al. (2015) | Gujarat, Uttar Pradesh, Bihar | (1) Trainings for ASHAs (and AWWs and rural medical practitioners (RMPs) on **diarrhea prevention and treatment**, involving written and pictorial educational materials and classes; (2) improved ORS and zinc supply chain. | | | Increasing knowledge of ORS and zinc improved management of childhood diarrhea among all three health worker cadres. ASHAs had highest rates of correctly advising ORS and zinc (ASHAs; 78%, AWWs: 65% and RMPs 20%) and more ASHAs had access to ORS and zinc (55%) compared to AWWs (39%) and RMPs (34%). Higher ORS/zinc knowledge scores were associated with correctly prescribing ORS and zinc among ASHAs and AWWs (aOR 2.48; 95% CI: 1.90-3.24) and RMPs (aOR: 2.32; 95% CI: 1.29-4.17). | | positive |
| Mazumder, Taneja, et al. (2014) | Haryana | **Integrated management of neonatal and child illness (IMNCI):** 3 year intervention involved: (1) training for CHWs (including ASHAs) and higher level health workers to improve IMNCI knowledge and skills; (2) CHWs conducting home visits to counsel mothers, and identify, refer and treat sick newborns; (3) strengthening of health systems with improved supervision and supply chain, and the addition of performance based incentives; and (4) increasing women’s awareness of services through village meetings. | | | Compared to the control areas, in the IMNCI intervention areas: appropriate careseeking was significantly higher for severe neonatal illness, local neonatal infection, and diarrhea, and pneumonia; there were fewer reported episodes of severe neonatal illness; lower prevalence of diarrhea and pneumonia; and infants were more likely to still be exclusively breastfed in the sixth month of life. | | positive |
| Prinja, Mazumder, et al. (2013) | Haryana | Cost analysis of the above **IMNCI** cluster randomized study. ANMs, AWWs and ASHAs were randomly selected from the intervention and control areas to collect cost data. | | | IMNCI imposes additional costs to the health system. The annual per-child cost of providing health services through an ANM, AWW and ASHA is INR 348 (USD $7.7), INR 588 ($13.1) and INR 87 ($1.90), respectively. The incremental cost of delivering IMNCI is INR 124.8 ($2.77), INR 26 ($0.60) and INR 31 ($0.70) at the ANM, AWW and ASHA level, respectively. Providing child health care services, with IMNCI, additionally cost INR 125 ($2.80), INR 26 ($0.60) and INR 83 ($1.80) at the ANM, AWW and ASHA level respectively. Major drivers of child health care costs at the AWC and ANM level were capital and human resources and at ASHA level human resources (57%) and drugs (32%) were significant cost drivers. | | NA |
| Das, Panwar, et al. (2014) | Uttar Pradesh | **Home based newborn care (HBNC)**: ASHA received a refresher training (5 days) that included orientation on HBNC guidelines, instruction on data recording formats, and an **IMNCI** skills review. | | | Overall, ASHAs did not complete comprehensive assessments. Using IMNCI’s color codes, ASHAs misclassified 80% of infants. ASHAs did not follow HBNC formats, skipped critical signs, and often failed to follow correct procedures for weighing and taking temperature, and failed to give full assessments for breastfeeding and cord health. Overall ASHA-investigator agreement on diagnosis was poor (kappa=0.23, P=0.01) and on the need for further assessment of infants was intermediate (kappa 0.48, P=<0.001). ASHAs need improved training, tools, and supportive supervision. | | negative |
| Panwar et al. (2012) | Uttar Pradesh | **Vistaar HBNC project:** Ongoing ASHA capacity building and supportive supervision to improve HBNC. ASHA monthly meetings were restructured, leading to more meetings but with smaller numbers of ASHAs (30-50 ASHAs at a time) on fixed days; structured content was developed for two-hour capacity building sessions covering IPC skills, delivering critical newborn care messages, use of job aids, planning home visits and organizing community meetings; block level facilitators were trained in the use of participatory methods and facilitation skills; ANMs were trained in supportive supervision; and technical resource groups were developed in districts for planning, implementation, and monitoring of the performance management support to ASHAs and ANMs. | | | ASHAs made home visits to 40% of all recently delivered women. Compared to project baseline, a significant increase was reported in the second newborn care visit (21% to 60%) and the third visit (8% to 40%) by ASHAs. Recently delivered women were able to recall newborn care messages given by ASHAs during antenatal ANC visits including, initiating immediate breastfeeding within one hour of birth, exclusive breastfeeding up to six months, newborn immunizations, and benefits of colostrum feeding. Messaging on keeping newborns warm, delaying bathing for seven days and not applying anything to the cord need further improvement.  Breastfeeding within one hour of birth improved from 10% at baseline to 27% in the end line. Colostrum feeding showed a 22% improvement over the baseline to reach 79%. Capacity building sessions have been held in 93% of ASHA monthly meetings. ASHA attendance has improved significantly at 74% in March 2012 compared to 48% in September 2009. | | positive |
| Tripathy, Nair, et al. (2016) | Odisha, Jharkhand | **Ekjut**: Participatory women's groups facilitated by ASHAs to improve maternal and newborn health. In the intervention group, ASHAs supported women’s groups through a participatory learning and action meeting cycle. Groups discussed and prioritized problems, identified and implemented strategies to address them, and assessed their progress. | | | The neonatal mortality rate was 30 per 1000 live births in the intervention group and 44 per 1000 live births in the control group (odds ratio [OR] 0.69, 95% CI 0·53–0·89). This reduction in neonatal mortality was largely driven by improvements in safe practices for home deliveries. The intervention’s impact was influenced by: (1) acceptability; (2) a participatory approach to the development of knowledge, skills and ‘critical consciousness’; (3) community involvement beyond the groups; (4) a focus on marginalized communities; (5) the active recruitment of newly pregnant women into groups; (6) high population coverage. | | positive |
| Mahanta, Islam, et al. (2016) | Assam | **HBNC incentive vouchers**: ASHAs received capacity building in HBNC and all mothers who delivered were given a set of 6 HBNC double perforated numbered vouchers which they handed over to ASHAs during home visits for new born care. ASHAs received their HBNC incentive only after the submission of all six vouchers. | | | The voucher system significantly improved HBNC practices, antenatal and post-natal care, iron folic acid supply, and immunization (p < 0.05). ASHA knowledge was assessed, with 100% having sufficient knowledge about immunization, 91% about young infant and child feeding, 82% about importance of home visits, 78% about family planning and reproductive tract infection, 77% about the importance of safe delivery, 68% birth preparedness and 55% essential newborn care. | | positive |
| Stalin, Krishnan, et al. (2011) | Haryana | All ASHAs (n = 33) linked to a PHC received training in **newborn care**, a weighing scale, refresher training at three and six months, and supportive supervision. | | | ASHA mean knowledge score (out of 11) increased from 6.45 pre-training to 6.50 immediately after training, 7.45 after 3 months, and 7.15 after six months. About 83% of the newborns born at home were weighed within 3 days of birth and 44% of ASHAs weighed the neonates within ±250 grams of the weight recorded by the author. | | mixed |
| Shashikala, Kulkarni, et al. (2016) | Karnataka | One-time training for frontline health workers (including ASHAs) on **breastfeeding and complementary feeding** | | All three educational interventions tested (information leaflets, lecture, and lecture with demonstration) significantly improved knowledge. Lecture with demonstration was considered most effective and feasible. | | | positive |
| Vir, Kalita, et al. (2014) | Chhattisgarh | **Nutrition Security Innovation Project**, a 3-year intervention to improve nutrition, embedded within the mitanin program. Mitanins (1) conducted frequent counseling to promote of exclusive breastfeeding and appropriate complementary feeding during pregnancy and child’s first year; (2) promoted kitchen gardens; and (3) informed community of entitlements to government subsidized food. | | While prevalence rates of underweight, stunting, and wasting among children were not significantly different between the intervention and control groups, the overall state annual average reduction rate for underweight was 4.22% and stunting was 5.64%. A higher percentage of intervention Mitanins than comparison mitanins visited their households in the preceding 30 days (92% v. 85%) spent more than 10 minutes with the families The proportion of mitanins who organized cluster meetings of community members with health functionaries was higher in the intervention than in the comparison areas (93.1% vs. 19.7%). | | | positive |
| Thakre, Thakre, et al. (2012) | Maharashtra | One-time training for ASHAs and supervisors on **breastfeeding and complementary feeding** | | The training significantly improved knowledge and skills. | | | positive |
| Modi, Patel, et al. (2016) | Gujarat | **ImTeCHO**: A mobile application with many ASHA support and monitoring functions. Links ASHA incentives to digital records, provides ASHAs regular feedback, helps them schedule visits, enables onsite data entry, seeks to increase demand generation among beneficiaries, supports health care decision making for ASHAs through algorithms. | | ImTeCHO was found to be acceptable, feasible, and useful, including for household enumeration and registration of pregnancies, deliveries and infant deaths. Adaptations to the intervention and its delivery included: (1) a helpline for ASHAs, (2) further simplification of ImTeCHO incentive management system; and (3) additional web-based features for enhancing value and supervision of Primary Health Center staff. | | | positive |
| Nair, Harikumaran Nair, et al. (2014) | Kerala | ASHAs received one day of training and then conducted community screening for **developmental delay and disability** among children | | Of the 101,438 children screened by ASHAs, 2,477 (2%) were referred to government developmental evaluation camps for having two or more delay items. Only 1,329 of these children reached evaluation camps, of which 572 (43%) were assessed to be developmentally normal (e.g., false positives). | | | NA |
| Raju (2012) | Karnataka | ASHAs were engaged to conduct a house-to-house survey in rural areas to assess the accuracy of **civil registration system statistics** | | The number of live births and deliveries reported by the local administration were higher than the number identified by the ASHAs. The number of stillbirths and maternal deaths matched. | | | positive |
| **Sexual and reproductive health, including maternal health (n=14)** | | | | | | | |
| Goel, Gupta, et al. (2013) | Haryana | **Nischay scheme**: health centers and ASHAs (n=15) were provided with Nischay kits (urine pregnancy test kits). ASHAs were trained in their use. Efforts were made to raise community awareness on home based pregnancy test card and RCH services; to empower rural women for early detection and timely registration of pregnancy. | There was no significant change in ANC registration in the six months after the launch of the Nischay scheme and only 16 % of beneficiaries were aware of the scheme. Of those who were aware, 40% learned about it from their ASHA while the remaining 60% learned about it from the ANM. Most (93%) of the ASHAs (14 of 15) had adequate knowledge on how to use the Nischay kit. | | | | negative |
| Derenzi, Wacksman, et al. (2016) | Uttar Pradesh | **ASTRA, an add-on to CommCare**: Within the Reducing Maternal and Newborn Deaths (ReMiND) intervention, ASHAs received and were trained to use the CommCare mobile phone application to guide their work and aid data collection. ASTRA was an added application with performance metrics to incentivize home visits to pregnant women. | ASHAs with access to the ASTA system made significantly more client visits, with average monthly visits 22% higher than ASHAs who had access to a control system. In addition, higher ASHA performance was correlated with increased usage of ASTA. However, the performance improvement was front-loaded, with the impact of the system decreasing toward the end of the study period. | | | | mixed |
| Deshpande, Bhanot & Maknikar (2015) | Maharashtra, Tamil Nadu, Rajasthan, Bihar | **Project Raksha**: sought to promote timely referral for obstetric complications through a multipronged social marketing intervention. ASHAs received training on technical aspects of maternal and newborn health and sought to persuade families to prepare for a safe birth, to organize transport, money, and potential blood donors; to recognize danger signs during pregnancy; and to access health facilities without delay. | Most (80%) of ASHAs reported regularly providing birth planning and complication readiness advice to pregnant women and their families. One third reported handling complications during pregnancy or delivery over the last two years by rushing women to health facilities. | | | | positive |
| Nyamathi (2014); Nyamathi, Ekstrand, et al. (2013); Nyamathi, Hanson, et al. (2012); Nyamathi, Salem, et al. (2013); Nyamathi, Sinha, et al. (2013) | Andhra Pradesh | **ASHA Life**: ASHAs were recruited, trained (for 3 days), supported, and monitored to provide **HIV/AIDS** knowledge, care, support group facilitation, nutritional supplementation and bus tokens to the hospital to rural women living with HIV/AIDS. | Women receiving the ASHA Life intervention showed significant changes compared to usual care group: improvement in ART adherence; reduction in barriers to ART; reduction in depressive symptoms, internalization of stigma and use of avoidant coping strategies; and improvement in CD4 counts, BMI, muscle mass and fat mass. | | | | positive |
| Gogoi, Parmar, et al. (2016) | Jharkhand | Training intervention: provided health care providers (including ASHAs) and adolescent married couples with information on **contraceptives and family planning** to delay first pregnancy. | The intervention increased awareness of condoms and copper-T/intrauterine device among married adolescents and increased their reported use of contraception. After the intervention, a greater portion of adolescents reported being advised by their ASHA or ANM to delay first pregnancy. | | | | positive |
| Sebastian, Khan, et al. (2012) | Uttar Pradesh | Behavior change communication intervention to promote **postpartum birth spacing**: health workers (ASHAs, ANMs and AWWs) received two days’ training plus communication materials (leaflets, posters, wall paintings and booklets) and educated pregnant women, husbands and mothers-in-law on pregnancy timing and spacing, antenatal and postnatal care. | Training significantly increased health worker knowledge on postpartum contraception from 14% answering all questions correctly to 95%. Compared to the control area, beneficiaries in the intervention area reported significantly higher rates of receiving counseling on contraception and spacing, an increase in knowledge and increase in use of modern contraceptive methods for spacing. | | | | positive |
| Johnston, Ganatra, et al. (2016) | Rajasthan (and South Africa and Ethiopia) | CHW training intervention (2 to 4 days) on **medical abortion** in Ethiopia, South Africa and India (ASHAs). CHWs were taught to use a urine pregnancy test, gestational age wheel, and checklist of screening questions related to the result of the urine pregnancy test, gestational age wheel assessment, date of last menstrual period, and 7 questions on contraindications to medical abortion. | When used by clinicians, the checklist toolkit was excellent at ruling out participants who were not eligible, and moderately effective at ruling in participants who were eligible for medical abortion. When used by CHWs, overall accuracy was 92% in Ethiopia, 80% in India and 77% in South Africa. Wrongly assessing participants as eligible for medical abortion remained a concern. | | | | positive |
| Singh (2009) | Jharkhand | Multi-pronged intervention to increase women’s knowledge of safe **medical abortion** services and community support for accessing abortion: Engaged community health workers (including Sahiyas (ASHAs) and outreach workers and used direct inter-personal communications, street theatre, and wall signs. | The average monthly client load in two public facilities and two private clinics that participated in the pilot initiative increased from 13 to 65 uterine evacuations per month from October 2008 to January 2009. | | | | positive |
| Bijalwan, Bhagavatula, et al. (2015) | Uttarakhand | ASHAs received a 1-day training on **uterine prolapse** screening then administered a screening questionnaire to women. | No findings were discussed on ASHA capacity to screen for uterine prolapse. It appears that all cases identified by ASHAs through screening were indeed prolapse cases. | | | | NA |
| Singh (2015) | North Indian state | ASHAs (n=250) trained (5 lectures) on **cervical cancer** screening conducted in a tertiary care hospital. | ASHA knowledge on cervical cancer screening improved by 25%. After training, most ASHAs wanted themselves (98%), their relatives (100%) and the woman within their area (98%) to be screened for cancer cervix. | | | | positive |
| **Communicable disease (n=6)** | | | | | | |  |
| Tiwari, Pandey & Chandra (2012) | Bihar | Workshop trained ASHAs to support people with **leprosy** and trained people with leprosy in self-care. | | | | After the workshops, 80% of planter ulcers healed. Secondary impairments were controlled and recurrence of ulcer was checked. | positive |
| Jonnalagada, Rao, et al. (2012) | Andhra Pradesh | ASHAs were trained to conduct a rapid survey to detect **leprosy** | | | | The 140 ASHAs detected 1223 suspected leprosy cases from a population of 98,834, which are awaiting validation. | NA |
| Das, Friedman, et al. (2015) | Odisha (Orissa) | Community mobilization, implemented by NGOs, to increase bed net use and appropriate **febrile illness** careseeking through community meetings, posters, leaflets, street plays, etc. While intervention arm A and B received community mobilization, arm A also received supportive supervision for the ASHAs (at least two supervisory visits per month from implementing NGO) on effective malaria case management. | | | | Significant improvements were observed in the reported utilization of bed nets in both intervention arms (84.5% in arm A and 82.4% in arm B versus 78.6% in the control arm; p < 0.001). While overall rates of treatment-seeking were equal across study arms, treatment-seeking from a CHW, treatment seeking from a skilled provider within 24 hours, and timely diagnosis of fever was higher in the intervention arms. | positive |
| Das, Pandey, et al. (2014); Das, Pandey, et al. (2016) | Bihar | One-time training for ASHAs on vector control, **Visceral Leishmaniasis**, post VL dermal leishmaniasis case identification and referral to PHCs for diagnosis and treatment. Training was provided during ASHA monthly meeting days by experts from the research team. In follow up on earlier study, ASHAs received additional training on detection and referral of VL. | | | | ASHA knowledge about VL increased significantly with training and this resulted in increased recruitment of patients for diagnosis and treatment to the local PHCs. At baseline, 7% of VL cases seeking treatment at the PHCs were referred by ASHAs. After one training, 28% of cases were referred by ASHAs. After two training sessions, they increased to 46%. Authors conclude that ASHA training is an effective way to conduct active case detection of VL cases and should be repeated once a year. | positive |
| Valadez, Devkota, et al. (2014) | Odisha (Orissa) | Lot Quality Assurance Sampling intervention wherein health system staff periodically monitored sub-district performance and used the findings to make improvements, such as ASHA knowledge, skills and supplies (rapid diagnostic tests, **Plasmodium falciparum** treatment) | | | | The LQAS results were able to support district managers to increase coverage in underperforming areas, especially for vertical strategies in the presence of diligent managers. Difference-in-differences tests showed that intervention districts exhibited significantly greater change in four of six vertical strategies (including insecticide treated bed-nets and indoor residual spraying), one of six treatment-seeking behaviors and four of 12 ASHA capacity indicators. The control district displayed greater improvement than two intervention districts for one ASHA capacity indicator. | positive |
| **Non-communicable disease (n=4)** | | | | | | |  |
| Menon, Joseph, et al. (2014) | Kerala | **ENDIRA (Epidemiology of Non-communicable Diseases in Rural Areas) study**: ASHAs were trained to conduct an NCD prevalence and risk factor survey, including taking height and weight measurements | | | | ASHAs were able to effectively conduct a prevalence study of NCDs, including prevalence of risk factors. There were high levels of agreement between ASHAs and physicians in data recorded in the questionnaires for myocardial infarction (agreement 95%; k = 0.80), stroke (91%; k 1= 0.85), hypertension (90%; k = 0.81) and diabetes (97%; k = 0.81). | positive |
| Jose, Pisharady, et al. (2015) | Kerala | **NCD control** pilot program wherein ASHAs conducted burden assessment, community education and follow up and nurses conducted screening for diabetes and hypertension. Continuous drug supplies were ensured. | | | | The community exhibited increased awareness about NCDs. Detection camps were over-crowded but all the patients who were examined by doctors were given medicines for one month and were followed up by ASHAs. Almost all ASHAs claimed 100% survey coverage but data quality varied. The study highlights that even with strong support from the NHRM in terms of funding and logistics, greater attention must be given to program management and clear guidelines for supervisors. | mixed |
| Armstrong, Kermode, et al. (2011) | Karnataka | Community health workers (n=20) including ASHAs (n=6) trained (4 days) in recognition of **mental disorders**, appropriate response and referral, support for people with mental disorders and their families, and mental health promotion in communities. | | | | The training course improved CHWs’ ability to recognize a mental disorder in a vignette, and reduced participants’ faith in unhelpful and potentially harmful pharmacological interventions. There was evidence of a minor reduction in stigmatizing attitudes, and it was unclear if the training resulted in a change in participants’ faith in recovery following treatment. | positive |
| Praveen & Patel (2014) | Andhra Pradesh | **SMARTHealth India** (Systematic Medical Appraisal Referral and Treatment in India): (1) a mobile device–based clinical decision support systems for CVD risk management, (2) task-shifting traditional physician roles to other health workers (ASHAs), and (3) integration of the overall system within government infrastructure. | | | | Of the 88 patients ASHAs referred (from 227 patient screened), 78% had a definite indication for blood pressure-lowering medication. However, despite high acceptability by end users, actual transformation was substantially limited by system-level barriers such as patient access to doctors and medicines. | mixed |
| **General ASHA work (n=5)** | | | | | | | |
| Fotso, Higgins-Steele & Mohanty (2015);  Elazan, Higgins-Steele, et al. (2016) | Odisha (Orissa) | **Male community health workers**, called male health activists (MHAs), were recruited, trained using the ASHA modules, and paired with ASHAs to improve coverage of RMNCH. | | | | MHAs were appreciated by ASHAs and other health workers for conducting outreach to remote areas, speaking to men about family planning, promoting antenatal care and neonatal care, supporting transportation during delivery, especially at night, and handling logistics at the health facility outside the labor and delivery room. Male CHWs would be a welcome compliment to ASHAs in the community-based delivery of, and increased demand for, RMNCH services. | positive |
| Kaphle & Chaturvedi (2015) | Bihar | **CommCare**: ASHAs and AWWs (n=600) were trained to use CommCare, a mobile tool to record and track RMNCH; register pregnancies, births and deaths; and help provide counseling. It provides decision support, aid in work planning and scheduling and has a reproductive health checklist to ensure comprehensive care. It provides multimedia to enhance ASHA-beneficiary communication. | | | | CommCare can positively impact the quality and experience of care provided by CHWs. The mean quality and experience among high CommCare adopters was 33% higher (p=0.04) than among lower adopters. Technology adoption was influenced by CHW literacy and age. | positive |
| Modi, Gopalan, et al. (2015) | Gujarat | **ImTeCHO**: A mobile application with many ASHA support and monitoring functions. Links ASHA incentives to digital records, provides ASHAs regular feedback, helps them schedule visits, enables onsite data entry, seeks to increase demand generation among beneficiaries, supports health care decision making for ASHAs through algorithms. | | | | This formative evaluation of a complex mHealth intervention found that the pilot intervention was largely acceptable, feasible, and useful. A few changes were made to the intervention and its delivery, including a new helpline for ASHAs, further simplification of processes within the ImTeCHO incentive management system and additional web-based features for enhancing value and supervision of Primary Health Center staff. | positive |
| Vashistha, Kumar, et al. (2016) | Uttar Pradesh | **Projecting Health**: mobile phone videos on maternal health were distributed through three different channels: mobile shop owners, laptop owners, and ASHAs. | | | | All three distribution channels were successful in targeting the community; mobile shops had access to most community members but ASHAs were most successful in getting videos out to those who were most interested in viewing them. Many participants were motivated to distribute the videos for the benefit of community. However, distribution rates decreased over time, suggesting the need for mechanisms to extrinsically motivate intermediaries and viewers for broader video distribution. | positive |

AWW: anganwadi worker; aOR: adjusted odds ratio; CI: confidence interval; IMNCI: Integrated Management of Neonatal and Childhood Illness; ORS: oral rehydration salts; RMP: rural medical practitioner (an unlicensed informal provider of health care)
