# Supplementary material for: Taking stock of 10 years of published research on the ASHA programme: examining India’s national community health worker programme from a health systems perspective
Source: Health Res Policy Syst. 2019 Mar 25;17:29. doi: 10.1186/s12961-019-0427-0 (PMC6434894; doi:10.1186/s12961-019-0427-0)
Supplement: Supplementary file 6 — Research on ASHAs, by typology and methodology. (DOCX 133 kb) [file 12961_2019_427_MOESM6_ESM.docx]

| **Additional file 6. Research on ASHAs, by typology and methodology** | |
| --- | --- |
| DESCRIPTIVE STUDIES: To describe for basic understanding and comparability without a predetermined framework, usually through a cross-sectional study (N=44) | |
| Mixed | - Surveys, FGDs and key informant interviews on selection, knowledge or performance of ASHAs, including utilisation by beneficiaries for general ASHA work [1], pre-eclampsia [2], newborn care [3], community-health service interface [4], immunization [5] |
|  | - Post intervention survey on effect of intervention to reduce obstetric emergency delays [6] |
|  | - System generated data and interviews (including with ASHAs) of health system readiness to treat malaria [7] |
|  | - Document review, interviews and observations of village health and nutrition days to understand perspectives and preparedness [8,9] |
|  | - Realistic evaluation of ASHA functionality and effectiveness in eight Indian states [10] |
| Qualitative | - Interviews and FGDs on selection, knowledge or performance of ASHAs, including utilisation by beneficiaries for general ASHA work and equity [11] and JSY [12] |
|  | - Formative evaluation with interviews, observations, and supervisory visit notes on mhealth MNCH intervention [13] |
| Quantitative | - Surveys on selection, knowledge or performance of ASHAs, including utilisation by beneficiaries on general ASHA work [14–17], effect of ASHA educational level [18–20], newborn care including observation of ASHAs on mannequins [21], child health [22], infant and young child feeding [23], ORS zinc with observation of ASHAs [24], maternity care [25], JSY [26,27], VL [28], tobacco [29], malaria [30,31], TB [32], mental illness [33], leprosy [34,35] |
|  | - Surveys by ASHAs to screen at population level, with studies verifying the accuracy of ASHA’s ability through additional data sources to assess: accuracy in detecting child illness verified by observation [36], accuracy to screen eligibility for medical abortion verified by observation [37], uterine prolapse [38], leprosy [39], NCDs [40], childhood disability through expert review [41], vital statistics verified by household surveys [42], mass drug administration for filiariasis [43], accuracy of mhealth registration verified by household survey [44] |
| INFLUENCE STUDIES: Evaluations that measure the effect of one variable on another (N=29) | |
| Adequacy or before and after evaluations (N=11) | |
| Mixed | - HMIS records, interviews and FGDs to assess JSY’s impact on maternal healthcare, out of pocket spending and ASHA performance motivation [45] - Pre- and post-intervention survey of a family planning intervention for married adolescent couples with qualitative provider interviews [46] - Retrospective analysis of health records to evaluate whether pregnancy detection kits increase ANC registration, combined with qualitative interviews [47] |
| Quantitative | - Pre- and post-training ASHA knowledge assessments on mental health [48], infant feeding [49], cervical cancer [50], newborn care (also checked accuracy of weighing) [51] - Before and after assessment of introducing home based neonatal care incentives [52] and improved support structures [53] on knowledge and practices, increasing knowledge of safe medical abortion on average monthly client load at health centers [54], increase in ASHAs on immunization coverage [55] |
| Plausibility: Evaluations with controls, but that are not randomized, with potential biases adjusted for in analysis (n=5) | |
| Mixed | - Comparison of regions based on IMNCI implementation data to assess IMNCI progress [56] |
| Quantitative | - Comparison of different durations of ASHA training on detection and referral for visceral leishmaniasis [57] - Urban-rural comparison of maternity indicators to evaluate the role of ASHA workers in rural areas [58] - Comparison of regions using Lot Quality Assurance Sampling on district-level malaria management [59] - Comparison of administrative blocks to assess child nutrition intervention [60] |
| Probability: Evaluations with randomized controls (n=13) | |
| Mixed | - Comparison of performance and motivation between ASHAs with and without the ASTA mobile application [61] - Cluster randomized control trial to evaluate participatory maternal health intervention [62] |
| Quantitative | - Cluster randomized control trials to evaluate cost effectiveness of IMNCI [63] and evaluate the effect of IMNCI [64,65], supportive supervision and community mobilization for malaria control [66], ASHA training for visceral leishmaniasis patient referrals [67], infant feeding [68], nutritional supplementation and counseling for HIV-related outcomes (ASHA-life intervention) [69–72], counseling by ASHAs and other frontline health workers on birth spacing [73] |
| EXPLORATORY STUDIES: Initial research to understand and build hypothesis, concepts and theories, often providing in-depth understanding of stakeholder perspectives (N=17) | |
| Mixed | - Surveys, in-depth interviews and consultations to understand underlying barriers to using emergency contraception [74] |
| Qualitative | - In-depth interviews and/or FGDs to explore underlying mechanisms of General ASHA work [75–77], barriers to point of care testing [78], JSY [79,80], potential of mhealth for cardiovascular health [81], NCD pilot [82] |
|  | - Ethnography of general ASHA work [83], immunization as modern Indian citizenship [84] |
|  | - Key informants, in-depth interviews, and FGDs using video presentations and case scenarios to understand factors underpinning treatment delays for child pneumonia [85] |
|  | - In-depth interviews to explore task-sharing with male health workers [86,87], coordination between ASHAs, ANMs and ASHAs [88] |
| Quantitative | - Development of a scale to measure communication and leadership [89] |
|  | - Development of a framework for mhealth adoption [90] |
| EXPLANATORY STUDIES: In-depth research using and testing theory to explain causal mechanisms, often with triangulation of data sources (N=23) | |
| Mixed | - Surveys with in-depth interviews and/or FGDs to understand the underlying mechanisms of the general ASHA program [91], mhealth for cardiovascular disease [92], dissemination of health videos on mobile phones [93], functioning of VHSNCs [94] |
|  | - Verbal autopsies, hospital records, site visits, document review to understand factors underpinning maternal deaths [95] |
|  | - Program monitoring data, document review, in-depth interviews and FGDs to understand scaling up of diarrhoea management [96] |
|  | - Likert scale survey and FGDs to measure and explain determinants of performance and motivation [97], survey with structural equation modelling and in-depth interviews to explain determinants of emotional labour [98] |
|  | - Self-answered questionnaires, household survey, in-depth interviews and focus group discussions on remuneration [99], survey and ethnography to understand the feminisation of labour [100] |
| Qualitative | - Case study research to understand underlying mechanisms of the general ASHA program [101–103] |
|  | - Ethnography of integration and teamwork [104], citizenship [105], incentivisation [106], community participation [107] |
| Reviews | - Reviews that draw from previous CHW programs in India to explain the underlying mechanisms of the ASHA program [108], including the rights of ASHAs [109] |
|  | - Reviews that draw from CHW programs elsewhere to understand CHW program elements of Immunization programs [110] |
|  | - Reviews that compare the ASHA program with other CHW programs to understand scale-up [111], remuneration [112] or systems integration [113] |
| REFLECTIVE COMMENTARIES (N=9) | |
| - Reflections on community processes recommended for the ASHA program [114] - Reflection on potential role of the ASHA for newborn care [115] - Commentary on ASHA-life HIV intervention [116] - Reflection on issues around CHW program scale up [117] - Critical reflections on training in NHRM [118], the NRHM and ASHA program [119–122] | |
